# Comparative Transcriptome Analysis of the Regenerating Zebrafish Telencephalon Unravels a Resource With Key Pathways During Two Early Stages and Activation of Wnt/β-Catenin Signaling at the Early Wound Healing Stage

**DOI:** 10.3389/fcell.2020.584604

**Published:** 2020-10-09

**Authors:** Yeliz Demirci, Gokhan Cucun, Yusuf Kaan Poyraz, Suhaib Mohammed, Guillaume Heger, Irene Papatheodorou, Gunes Ozhan

**Affiliations:** ^1^İzmir Biomedicine and Genome Center (IBG), Dokuz Eylül University Health Campus, İzmir, Turkey; ^2^İzmir International Biomedicine and Genome Institute (IBG-İzmir), Dokuz Eylül University, İzmir, Turkey; ^3^European Molecular Biology Laboratory – European Bioinformatics Institute (EMBL-EBI), Cambridge, United Kingdom; ^4^École Centrale de Nantes, Nantes, France

**Keywords:** brain regeneration, telencephalon, comparative transcriptome analysis, zebrafish, Wnt/β-catenin pathway, wound healing

## Abstract

Owing to its pronounced regenerative capacity in many tissues and organs, the zebrafish brain represents an ideal platform to understand the endogenous regeneration mechanisms that restore tissue integrity and function upon injury or disease. Although radial glial and neuronal cell populations have been characterized with respect to specific marker genes, comprehensive transcriptomic profiling of the regenerating telencephalon has not been conducted so far. Here, by processing the lesioned and unlesioned hemispheres of the telencephalon separately, we reveal the differentially expressed genes (DEGs) at the early wound healing and early proliferative stages of regeneration, i.e., 20 h post-lesion (hpl) and 3 days post-lesion (dpl), respectively. At 20 hpl, we detect a far higher number of DEGs in the lesioned hemisphere than in the unlesioned half and only 7% of all DEGs in both halves. However, this difference disappears at 3 dpl, where the lesioned and unlesioned hemispheres share 40% of all DEGs. By performing an extensive comparison of the gene expression profiles in these stages, we unravel that the lesioned hemispheres at 20 hpl and 3 dpl exhibit distinct transcriptional profiles. We further unveil a prominent activation of Wnt/β-catenin signaling at 20 hpl, returning to control level in the lesioned site at 3 dpl. Wnt/β-catenin signaling indeed appears to control a large number of genes associated primarily with the p53, apoptosis, forkhead box O (FoxO), mitogen-activated protein kinase (MAPK), and mammalian target of rapamycin (mTOR) signaling pathways specifically at 20 hpl. Based on these results, we propose that the lesioned and unlesioned hemispheres react to injury dynamically during telencephalon regeneration and that the activation of Wnt/β-catenin signaling at the early wound healing stage plays a key role in the regulation of cellular and molecular events.

## Introduction

All vertebrate brains studied so far undergo adult neurogenesis, where they generate functional new neurons and integrate them into the existing brain circuitry after fetal and early postnatal development. Adult neurogenesis is achieved *via* stem or progenitor cells, in particular zones that exhibit varying degrees of abundance and neurogenic capacity among vertebrate species, and correlates with the capacity to regenerate upon damage to neuronal networks caused by injury (Doetsch and Scharff, 2001; Chapouton et al., 2007; Kaslin et al., 2008; Bonfanti and Peretto, 2011; Alunni and Bally-Cuif, 2016). Radial glia or glia-like cells (RGCs) have the characteristics of astrocytes, the star-shaped glial cells of the central nervous system (CNS), and act as primary neural stem cells (NSCs) that generate new neurons in the adult brain (Doetsch et al., 1999; Noctor et al., 2001; Seri et al., 2001; Dhaliwal and Lagace, 2011; Ming and Song, 2011).

Mammalian adult neurogenesis takes place predominantly *via* the NSCs confined to two distinct parts of the forebrain, i.e., the subventricular zone (SVZ) of the lateral ventricles in the telencephalon and the subgranular zone (SGZ) of the dentate gyrus in the hippocampus (Alvarez-Buylla et al., 2001; Kriegstein and Alvarez-Buylla, 2009; Bonfanti and Peretto, 2011). The restricted capacity of the neurogenic niches is explained partially by the reduction of constitutively active adult proliferation zones in mammalian brain during evolution (Rakic, 2002; Lindsey and Tropepe, 2006; Tanaka and Ferretti, 2009), a specific or general resistance against cell proliferation due to the evolution of tight control mechanisms against tumorigenesis (Pearson and Sanchez Alvarado, 2008), resistance to integrate new cells into a mature neural network (Kempermann et al., 2004; Kaslin et al., 2008), an altered cellular plasticity that affects stem or progenitor cell characteristics (Shihabuddin et al., 2000; Peng et al., 2002; Kizil et al., 2012b), or a non-permissive environment related to scar formation at the wound site after injury (Ekdahl et al., 2003; Fitch and Silver, 2008; Rolls et al., 2009). Low neurogenecity is paralleled by a limited potential in the integration of the newborn neurons in most regions of the mammalian brain, necessitating the use of another platform free of these constraints for the development of new therapeutic approaches (Bhardwaj et al., 2006; Ernst and Frisen, 2015; Alunni and Bally-Cuif, 2016).

In contrast to mammals, adult neurogenesis in zebrafish is robust and widespread, with 16 distinct constitutively proliferative zones that are mostly located along the ventricular surface but also deeper in the brain parenchyma (Kaslin et al., 2008). These zones are detected along the entire rostro-caudal axis of the zebrafish brain and contain ventricularly positioned self-renewing neural progenitors (Grandel et al., 2006; Kaslin et al., 2008; Kizil et al., 2012b; Grandel and Brand, 2013). Zebrafish CNS regenerates *via* the proliferation and differentiation of the radial glial cells (RGCs), also referred to as ependymoglia due to their functional orthology to the mammalian ependymal cells, and the neuroepithelial-like progenitor cells (Grandel et al., 2006; Ganz et al., 2010; Fleisch et al., 2011; Kizil et al., 2012b; Kaslin et al., 2017; Lindsey et al., 2017; Zambusi and Ninkovic, 2020). The permissive environment, which is necessary for these progenitors—upon injury—to become activated and differentiate into functional neurons, results most likely from the fact that zebrafish does not form any obvious scar tissue after injury in the CNS, thus granting zebrafish a regeneration potential higher than that of mammals (Becker and Becker, 2002; Kroehne et al., 2011).

Being one of the best-characterized regions of the adult zebrafish brain, the telencephalon harbors at least two distinct neurogenic zones, the dorsal and the medio-ventral neurogenic niches, with ependymoglial cells that differ in their proliferation and progeny characteristics (Marz et al., 2010; Zambusi and Ninkovic, 2020). Accordingly, these cells express different markers including glial fibrillary acidic protein (Gfap), S100β, nestin, brain lipid-binding protein (Blbp), aromatase, and SRY-box 2 (Sox2), confirming their radial glia-like nature (Adolf et al., 2006; Pellegrini et al., 2007; Lam et al., 2009; Ganz et al., 2010; Marz et al., 2010; Kroehne et al., 2011). A recent study on transcriptome analysis has identified novel markers for radial glia, newborn neurons, and mature neurons (Lange et al., 2020). Several inflammation-related programs including the chemokine receptor Cxcr5, the zinc finger transcription factor Gata3, and cysteinyl leukotriene signaling have been shown to promote the proliferation and generation of newborn neurons, thus enhancing neuronal regeneration in response to injury (Kizil et al., 2012a,b; Kyritsis et al., 2012). Fibroblast growth factor (Fgf) signaling and aryl hydrocarbon receptor (AhR) signaling have also been reported to directly regulate ependymoglial cell proliferation in the adult zebrafish telencephalon (Ganz et al., 2010; Di Giaimo et al., 2018). Yet, little is known about the neural signaling pathways and the molecular interaction networks that are involved in zebrafish brain regeneration. This is particularly in question for the early stages of brain regeneration, where a deeper knowledge of the contributory pathways with a special focus on their crosstalk is necessary to understand the underlying mechanisms.

To address these questions, we have set out to identify the genes that are differentially expressed in the zebrafish telencephalon during early regeneration in response to stab wound injury and focused on two early stages: (1) the early wound healing stage at 20 h post-lesion (hpl) and (2) the early proliferative stage at 3 days post-lesion (dpl). To this end, we have performed whole transcriptome analyses of the telencephalon by comparing (a) the lesioned and unlesioned hemispheres at two regenerative stages separately and (b) the lesioned hemispheres of the two stages to each other. Upon comparing the injury responses of the hemispheres at the early wound healing stage, we have found that the number of differentially expressed genes (DEGs) in the lesioned hemisphere is by far higher than that of the unlesioned hemisphere. In the early proliferative stage, however, the difference between the responses of the hemispheres declined noticeably and more than 40% of all DEGs are shared. Our comparative transcriptome analyses of the lesioned hemispheres at the wound healing and proliferative stages have revealed a considerably large pool of DEGs that are not shared, reflecting a discrepancy in the transcriptional profiles of these stages. Using Gene Ontology (GO) term and Kyoto Encyclopedia of Genes and Genomes (KEGG) pathway analysis, we have deciphered an enrichment of the gene sets related to the Wnt/β-catenin signaling pathway predominantly at 20 hpl and confirmed this experimentally at the messenger RNA (mRNA) and protein levels. To further investigate how Wnt/β-catenin signaling regulates regeneration at the molecular level, we have unveiled the Wnt targetome by analyzing the transcriptomes of the lesioned hemispheres where Wnt signaling is suppressed from the time of injury to 20 hpl or 3 dpl. Our data have demonstrated that the Wnt targetome at 20 hpl is considerably larger than that at 3 dpl and that Wnt/β-catenin signaling controls a variety of key signaling pathways including p53, apoptosis, forkhead box O (FoxO), mitogen-activated protein kinase (MAPK), and mammalian target of rapamycin (mTOR) at the early wound healing stage of brain regeneration. Overall, by using a prominent combination of bioinformatics and experimental validation of the bioinformatics data, we unravel the whole transcriptomes of the lesioned and unlesioned hemispheres at the early wound healing and proliferative stages of brain regeneration and highlight the significance of Wnt/β-catenin signaling in the regulation of cellular and molecular events taking place during early regeneration.

## Materials and Methods

### Stab Wound Assay

A transgenic reporter of Tcf/Lef-mediated transcription 6xTCF/Lef-miniP:2dGFP [Tg(6XTCF:dGFP)] (Shimizu et al., 2012) was used for RNA isolation and transcriptome analysis. Wild-type (wt) AB zebrafish were used for immunofluorescence staining. Before surgery, fish were anesthetized with 0.02% of Tricaine (MS-222, Sigma-Aldrich). Stab injury was performed in 6–9-months-old male fish as previously described (Kroehne et al., 2011; Baumgart et al., 2012). A 30-gauge needle was inserted through the left nostril until the end of the telencephalon. Afterward, the fish were transferred into a tank with freshwater. Animal experiments were approved by the Animal Experiments Local Ethics Committee of İzmir Biomedicine and Genome Center (IBG-AELEC).

### Drug Treatment

To inhibit Wnt/β-catenin signaling during regeneration, zebrafish that are exposed to stab wound injury of the brain were immersed in fish water containing 20 μM IWR-1 (Sigma-Aldrich, MO, United States) dissolved in dimethyl sulfoxide (DMSO). Inhibitor of Wnt response (IWR) treatment was initiated directly after stab injury and terminated at either 20 hpl or 3 dpl. IWR-1 containing fish water was refreshed daily.

### Tissue Preparation

After performing stab wound injury in the telencephalon, the fish were euthanized and severed heads were fixed in 4% paraformaldehyde (PFA) overnight at 4°C. Next, the heads were transferred into 20% sucrose–20% EDTA for 2 days at 4°C and 30% sucrose–20% EDTA overnight at 4°C. The heads were embedded in 20% sucrose–7.5% gelatin solution kept in a box filled with dry ice. Brain sections of 14 μm were cut at −20°C using a cryostat (CM1850, Leica, Wetzlar, Germany) and stored at -20°C until further use.

### Immunohistochemistry and Imaging

The brain sections on slides were dried at room temperature (RT) for 20 min. The slides were washed twice with PBSTX (1× PBS/0.3% Triton X-100) for 10 min. After incubation with 10 mM sodium citrate for 15 min at 85°C, they were washed with 1 × PBS twice for 5 min and incubated with diluted primary antibody in PBSTX at 4°C overnight. The next day, the slides were washed three times with PBSTX for 10 min at RT, incubated with secondary antibody, and washed three times with 1× PBS for 5 min at RT. Images were obtained using an LSM 880 laser scanning confocal microscope (Carl Zeiss AG, Oberkochen, Germany). The primary antibodies are as follows: mouse anti-GFAP (1:500, ab154474, Abcam, Cambridge, United Kingdom), mouse anti-PCNA (1:500, M0879, Dako, Agilent, CA, United States), rabbit anti-S100β (1:500, Z0311, Dako, Agilent), mouse anti-acetylated tubulin (1:250, T6793, Sigma-Aldrich, MO, United States), and rabbit anti-phospho-β-catenin (1:100, D2F1, Cell Signaling Technology, MA, United States). The secondary antibodies are as follows: rhodamine (TRITC) AffiniPure donkey anti-rabbit IgG (1:200, 711-025-152, Jackson Immunoresearch Laboratories, PA, United States) and Cy5 AffiniPure donkey anti-rat IgG (H+L) (1:400, 712-175-150, Jackson Immunoresearch Laboratories). Nuclear staining was performed with 4′,6-diamidino-2-phenylindole (DAPI; 4083S, Cell Signaling Technology).

### RNA Isolation and cDNA Preparation

**FIGURE 1:**
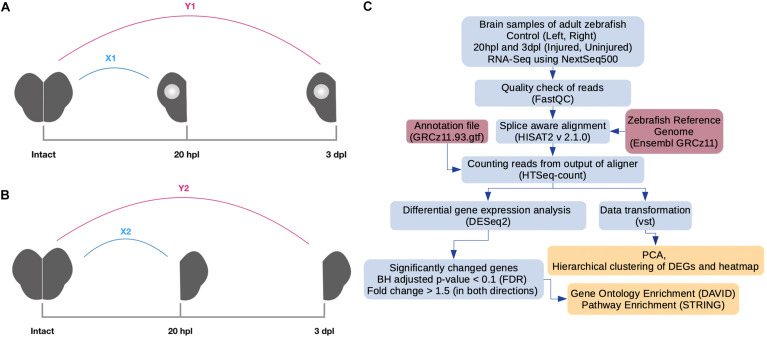
Pipeline for RNA preparation and analysis of RNA sequencing (RNA-seq) data. **(A)** Preparation of RNA samples from the lesioned hemispheres at 20 hpl or 3 dpl. *X1*: comparison of the lesioned hemisphere at 20 hpl with the intact brain; *Y1*: comparison of the lesioned hemisphere at 3 dpl with the intact brain. **(B)** Preparation of RNA samples from the unlesioned hemispheres at 20 hpl or 3 dpl. *X2*: comparison of the unlesioned hemisphere at 20 hpl with the intact brain; *Y2*: comparison of the unlesioned hemisphere at 3 dpl with the intact brain. **(C)** RNA-seq reads were generated using Illumina NextSeq 500. The quality of reads was checked by the FastQC tool. Next, alignment and counting of reads were performed by using a zebrafish reference genome (Ensembl, GRCz11) and the annotation file (Ensembl, GRCz11.93) by HISAT2 and HTSeq-count tools, respectively. Counts were transformed with variance stabilizing transformation (vst) to perform principal component analysis and hierarchical clustering. To select genes as differentially expressed, we used the DESeq2 package with the following criteria: fold change > 1.5 (in both directions) and Benjamini–Hochberg adjusted *p*-value (FDR) < 0.1. Gene Ontology and pathway enrichment analyses were performed by using DAVID and the STRING tool of Cytoscape, respectively. *hpl*, hours post-lesion; *dpl*, days post-lesion.

After stab wound injury was performed, zebrafish were anesthetized using 0.02% Tricaine-S solution and sacrificed at either 20 h post-lesion (hpl) or 3 days post-lesion (dpl). Firstly, the heads were separated from the fish and then the lesioned (left) and unlesioned (right) hemispheres were dissected individually to examine the influence of the injury between them (Figures 1A,B). The left and right hemispheres of healthy zebrafish telencephalon were used as control samples. The experiments were carried out in triplicate for each group. All brain samples were transferred into an RNAprotect tissue reagent (Qiagen, Hilden, Germany) immediately to prevent RNA degradation. Total RNA was extracted using the RNeasy Plus Micro Kit (Qiagen) according to the manufacturer’s instructions. RNA samples were quantified by using a NanoDrop 2000 spectrophotometer (Thermo Scientific, MA, United States). RNA integrity was assessed using an Agilent RNA 6000 Pico with the Agilent 2100 Bioanalyzer (Agilent Technologies, CA, United States). The samples were sent to the Genomics Core Facility (GeneCore, EMBL Heidelberg, Germany) for library preparation and RNA sequencing.

### Library Preparation and RNA Sequencing

Libraries were prepared using an Illumina TruSeq RNA Library Preparation Kit v2 (Illumina, San Diego, CA, United States) according to the manufacturer’s recommendations by using 500 ng complementary DNA (cDNA) as input. For size selection, 300 bp cDNAs were selected. Paired-end, strand-specific sequencing for total RNA was performed on Illumina NextSeq 500 with 75 bp read lengths.

### Bioinformatics Analysis

For each sample, quality control of the reads was inspected by using the FastQC tool; low-quality reads and adapter sequences were removed by using Trimmomatic (Bolger et al., 2014). RNA sequencing (RNA-seq) reads were aligned to the zebrafish reference genome (Ensembl, GRCz11) using HISAT2 (2.1.0) (Kim et al., 2015). After alignment, the transcripts were assembled and counted by HTSeq (Anders et al., 2015) by using the annotation file from the Ensembl website (Danio_rerio.GRCz11.93.gtf) (Figure 1C). The DESeq2 package (Love et al., 2014) of Bioconductor (Gentleman et al., 2004) was used to carry out normalization of read counts, their transformation (vst), and differential expression analysis. Principal component analysis (PCA) was performed to check the vst-transformed read counts and visualized with ggplot2 (Wickham, 2016; Supplementary Figure S1). To find DEGs, we performed Wald tests for the following comparisons using DESeq2: 20 hpl lesioned hemisphere vs. control (X1); 20 hpl unlesioned hemisphere vs. control (X2); 20 hpl lesioned hemisphere after IWR treatment vs. 20 hpl lesioned hemisphere (X3); 20 hpl lesioned hemisphere after IWR treatment vs. control (X4); 3 dpl lesioned hemisphere vs. control (Y1); 3 dpl unlesioned hemisphere vs. control (Y2); 3 dpl lesioned hemisphere after IWR treatment vs. 3-dpl lesioned hemisphere (Y3); and 3 dpl lesioned hemisphere after IWR treatment vs. control (Y4) (Figures 1A,B, 6B). For all eight groups, we selected a gene as upregulated if the fold change > 1.5 and downregulated if the fold change < 0.67 ( = 1/1.5), which we will simply refer to as “FC > 1.5 in both directions” hereafter (Supplementary Table S1). The lists of the significantly downregulated and upregulated genes obtained from individual comparisons were used as inputs for statistical enrichment analyses with regard to GO terms by using the Database for Annotation, Visualization and Integrated Discovery (DAVID) 6.8 bioinformatics functional annotation tool (Huang da et al., 2009). Functional enrichment was performed in three categories of GO terms: biological process (BP), molecular function (MF), and cellular component (CC). The EASE score, a modified one-tailed Fisher’s exact test, was used to identify the GO terms by means of a user-defined gene list for each defined DAVID GO term. KEGG pathway (Kanehisa and Goto, 2000) and Reactome pathway (Fabregat et al., 2018) analyses were performed using all significantly changed genes with the StringApp tool of Cytoscape 3.7.2 (Shannon et al., 2003; Doncheva et al., 2019). Gene lists related to the selected GO terms and KEGG pathways were obtained from the KEGG and AmiGO databases and plotted using the GOplot package (Walter et al., 2015).

### qPCR and Statistical Analysis

All samples with RIN ≥ 7.0 (our samples ranged from 8.1 to 10) were converted to cDNA using ProtoScript II First Strand cDNA Synthesis Kit (New England BioLabs, MA, United States) according to the manufacturer’s instructions. Zebrafish *rpl13a* (*ribosomal protein L13a*) was used as the reference gene for normalization to determine the relative gene expression levels. Quantitative PCR (qPCR) was performed in triplicate using GoTaq qPCR Master Mix (Promega, Madison, WI, United States) at an Applied Biosystems 7500 Fast Real Time PCR machine (Foster City, CA, United States). The data were analyzed using the GraphPad Prism 8 software (Graphpad Software Inc., CA, United States). The values are the mean ± SEM (standard error of mean) of three samples. Primer sequences for the following zebrafish genes are listed in Supplementary Table S2: *adam8a*, *c1qc*, *capgb*, *cd74b*, *cdk2*, *csf1ra*, *ctgfa*, *egfp*, *epha2a*, *foxo1a*, *fsta*, *gadd45ga*, *gfap*, *grem1b*, *il6st*, *inhbab*, *isl1*, *klf11b*, *lef1*, *mCherry*, *mpeg1.1*, *pappaa*, *pcna*, *prg4b*, *rpl13a*, *sgk2b*, *tbx2a*, and *tfa*.

## Results

### Transcriptome Profiling of the Telencephalon During the Early Wound Healing Stage of Regeneration

**FIGURE 2:**
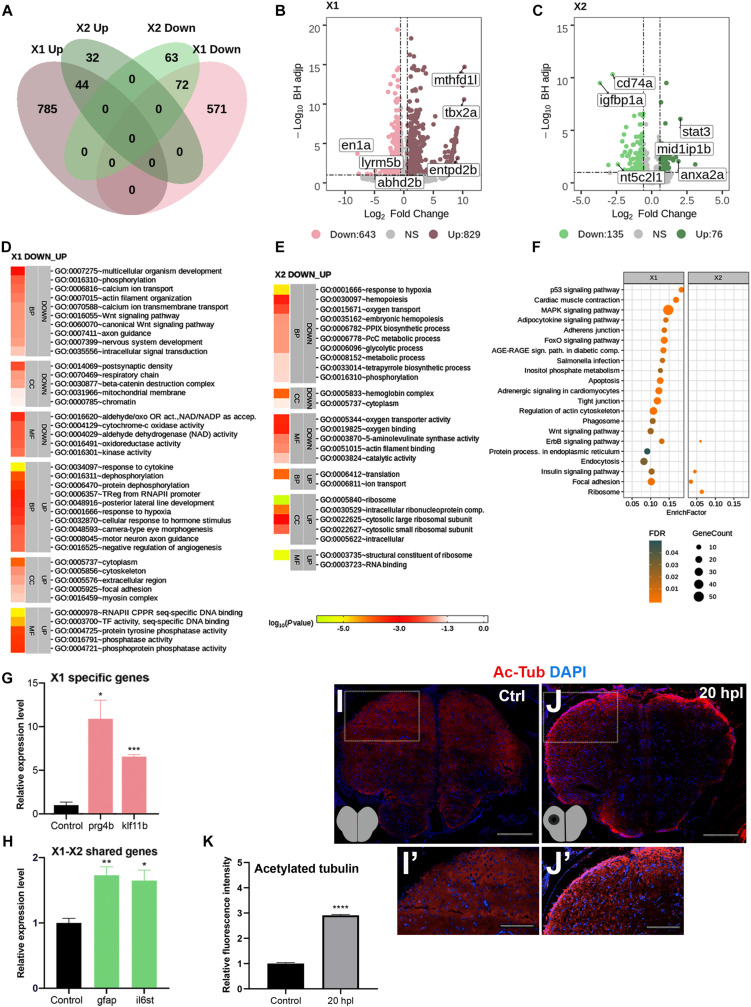
Transcriptome profiling of the telencephalon during the early wound healing stage of regeneration. **(A)** The Venn diagram shows the number of upregulated (*Up*) or downregulated (*Down*) differentially expressed genes (DEGs) in the lesioned (*X1*, *pink*) and unlesioned (*X2*, *green*) hemispheres and the overlap between each set of DEGs at 20 hpl. A total of 1,472 and 211 genes were significantly changed in X1 and X2, respectively. There were 116 genes shared between X1 and X2. **(B,C)** Volcano plots representing changes in the gene expression levels in X1 and X2. *Each point* represents a gene. The *X*-axis shows log_2_ of fold change in the condition compared to control and the *vertical dashed lines* indicate the fold change cutoffs. The *Y*-axis represents -log_10_ of the Benjamini–Hochberg adjusted *p-*value, where the significance threshold is indicated by the *dashed horizontal line*. Number of upregulated genes in X1 and X2: 829 and 76, respectively, and downregulated genes in X1 and X2: 643 and 135, respectively. *Darker* and *lighter shades* in **(A–C)** indicate upregulation and downregulation, respectively. **(D,E)** DAVID bioinformatics tool was used to show the most significantly enriched GO terms based on the transcriptional changes in X1 and X2 comparisons. The heatmap scale shows log_10_ of the EASE *p*-value for the most significantly enriched GO terms. **(F)** STRING bioinformatics tool was used to show the most significantly enriched KEGG pathways based on the transcriptional changes in X1 and X2. The *dot plot* represents KEGG pathways enriched in X1 and X2 by using all significantly changed genes in these comparisons based on FDR and EnrichFactor. **(G,H)** Relative expression levels of X1-specific genes (*prg4b* and *klf11b*) and the genes shared between X1 and X2 (*gfap* and *il6st*). Statistical significance was evaluated using unpaired *t*-test. *p < 0.05, **p < 0.01, and ***p < 0.001. *Error bars* represent ± standard error of the mean (SEM, *n* = 3). **(I–I′,J–J′)** Anti-acetylated tubulin staining of the control and 20 hpl brain sections with *boxed* areas magnified. Sections are counterstained for DAPI. *Scale bars*, 200 μm in **(I,J)**; 100 μm in **(I′)** and **(J′)**. **(K)** Relative fluorescence intensity in brain sections stained for anti-acetylated tubulin. *DAVID*, Database for Annotation, Visualization and Integrated Discovery; *GO*, Gene Ontology; *BP*, biological process; *CC*, cellular component; *MF*, molecular function; *STRING*, Search Tool for the Retrieval of Interacting Genes/Proteins; *KEGG*, Kyoto Encyclopedia of Genes and Genomes; *FDR*, false discovery rate; *hpl*, hours post-lesion. See section “Materials and Methods” for the definition of DEGs covered in X and Y comparisons. *p < 0.05, **p < 0.01, ***p < 0.001, and ****p < 0.0001.

While the later stages of brain regeneration have been relatively better understood, little is known about the early stages especially at the level of differential gene expression. To determine the changes in gene expression that control the molecular mechanisms underlying early brain regeneration, we aimed to identify and compare the entire transcriptomes of the zebrafish telencephalon at two early regenerative stages, i.e., 20 hpl and 3 dpl. Several telencephalon genes have been found to be significantly upregulated in the lesioned and unlesioned hemispheres (Kroehne et al., 2011; Kizil et al., 2012b). However, their injury responses have not been compared at the transcriptome level before. Thus, to distinguish between the responses of the lesioned and unlesioned hemispheres and avoid the dilution of genes that are differentially expressed mainly in the lesioned hemisphere, we decided to dissect the telencephalic hemispheres and have their transcriptome sequenced separately (Figure 1). Before sequencing, we validated the injury response by measuring the expression levels of *gfap* and *gata3* that are known to be strongly upregulated in the lesioned hemisphere upon injury (Supplementary Figure S1) (Kroehne et al., 2011). We found that 1,472 genes [829 upregulated (Up) and 643 downregulated (Down)] were significantly differentially expressed in the lesioned hemisphere at 20 hpl (X1) as compared to the equivalent hemisphere of the unlesioned brain, and 1,356 (785 Up and 571 Down) of them were unique to X1 (Figure 2A and Supplementary Table S3). *en1a*, *abhd2b*, and *lyrm5b* were in the top 10 Down genes while *mthfd1l*, *tbx2a*, and *entpd2b* were in the top 10 Up genes in X1 (Figure 2B). In the unlesioned hemisphere at 20 hpl (X2), we detected 211 DEGs (76 Up and 135 Down) with lower fold changes in both the Up and Down genes as compared to X1, and 95 genes (32 Up and 63 Down) were unique to X2 (Figure 2A and Supplementary Table S3). *igfbp1a*, *cd74a*, and *nt5c2l1* were the top three Down genes while *stat3*, *anxa2a*, and *mid1ip1b* were the top three Up genes in X2 (Figure 2C). Only 7% of all DEGs, i.e., 116 genes (44 Up and 72 Down), including those encoding for several heat shock proteins, leucine-rich repeat-containing proteins, and phosphatases were shared between X1 and X2 (Figure 2A and Supplementary Table S3). The top GO terms in X1 and X2 showed no overlap, parallel to the low number of genes shared between them (Figures 2D,E). We also determined altered GO terms and Reactome pathways by comparing the X1 and X2 groups (Supplementary Figures S2, S3 and Supplementary Tables S4, S5). While there were 20 KEGG pathways significantly enriched in X1, only four pathways were enriched in X2 (Figure 2F and Supplementary Table S5). To confirm the results of the bioinformatics analyses, we quantified the expressions of several genes related to tissue regeneration (Kroehne et al., 2011; Tsujioka et al., 2017; Galvao et al., 2018; Yu et al., 2019). *prg4a* and *klf11* were indeed upregulated only in the lesioned hemisphere (Figure 2G), while *gfap* and *il6st* were so in both hemispheres (Figure 2H). Several tubulin genes (*tubb6*, *tuba1b*, *tuba8l*, *tub*, and *tubb4b*) were upregulated not only in X1 but also in X2 (*tuba8l4*) (Supplementary Table S1). We confirmed this by immunofluorescence staining for acetylated tubulin, a neuronal microtubule marker that mainly labeled the parenchyma cells in the control and became strongly upregulated in the cells lining the ventricle after injury (Figures 2I,J). These data collectively indicate that, at the early wound healing stage, remarkably, i.e., seven times, higher numbers of genes are differentially expressed in the lesioned hemisphere as compared to the unlesioned hemisphere.

### Transcriptome Profiling of the Telencephalon During the Early Proliferative Stage of Regeneration

**FIGURE 3:**
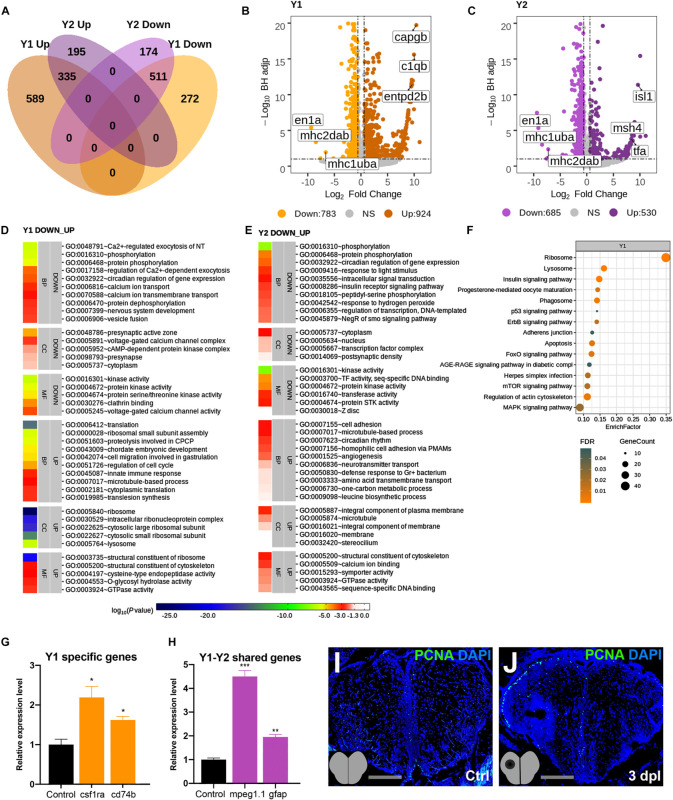
Transcriptome profiling of the telencephalon during the early proliferative stage of regeneration. **(A)** The Venn diagram shows the number of upregulated (*Up*) or downregulated (*Down*) differentially expressed genes (DEGs) in the lesioned (*Y1*, *orange*) and unlesioned (*Y2*, *purple*) hemispheres and the overlap between each set of DEGs at 3 dpl. A total of 1,707 and 1,215 genes were significantly changed in Y1 and Y2, respectively. There were 846 genes shared between Y1 and Y2. **(B,C)** Volcano plots representing changes in the gene expression levels in Y1 and Y2. *Each point* represents a gene. The *X*-axis shows log_2_ of fold change in the condition compared to the control and *dashed vertical lines* indicate the fold change cutoff. The *Y*-axis represents -log_10_ of the Benjamini–Hochberg adjusted *p*-value, where the significance threshold is indicated by the *dashed horizontal line*. Number of upregulated genes in Y1 and Y2: 924 and 530, respectively, and downregulated genes in Y1 and Y2: 783 and 685, respectively. *Darker* and *lighter shades* in **(A–C)** indicate upregulation and downregulation, respectively. **(D,E)** DAVID bioinformatics tool was used to show the most significantly enriched GO terms based on the transcriptional changes in Y1 and Y2 comparisons. The heatmap scale shows log_10_ of the EASE *p*-value for the most significantly enriched GO terms. **(F)** STRING bioinformatics tool was used to show the most significantly enriched KEGG pathways based on the transcriptional changes in X1 and X2. The *dot plot* represents KEGG pathways enriched in X1 and X2 by using all significantly changed genes in these comparisons based on FDR and EnrichFactor. **(G,H)** Relative expression levels of Y1-specific genes (*csf1ra* and *cd74b*) and the genes shared between Y1 and Y2 (*mpeg1.1* and *gfap*). Statistical significance was evaluated using unpaired *t*-test. *p < 0.05, **p < 0.01, and ***p < 0.001. *Error bars* represent ± standard error of the mean (SEM, *n* = 3). **(I,J)** Anti-PCNA staining of the control and 3 dpl brain sections. Sections are counterstained for DAPI. *DAVID*, Database for Annotation, Visualization and Integrated Discovery; *GO*, Gene Ontology; *BP*, biological process; *CC*, cellular component; *MF*, molecular function; *STRING*, Search Tool for the Retrieval of Interacting Genes/Proteins; *KEGG*, Kyoto Encyclopedia of Genes and Genomes; *FDR*, false discovery rate; *dpl*, days post-lesion. See section “Materials and Methods” for the definition of DEGs covered in X and Y comparisons.

The telencephalon is known to react to injury by a strong induction of proliferation that peaks at 3 dpl mainly in the ventricular zone of the lesioned hemisphere (Kroehne et al., 2011; Marz et al., 2011; Lindsey et al., 2017). By analyzing the transcriptomics data at 3 dpl, we identified 1,707 genes (924 Up and 783 Down) to be significantly differentially expressed in the lesioned hemisphere at 3 dpl (Y1) as compared to the corresponding hemisphere of the unlesioned brain, and 861 (589 Up and 272 Down) of them were unique to Y1 (Figure 3A and Supplementary Tables S1, S3). In contrast to 20 hpl, most DEGs were shared between Y1 and Y2 (unlesioned hemisphere at 3 dpl), where we identified a total of 1,215 DEGs (530 Up and 685 Down), only 369 (195 Up and 174 Down) of which were unique to Y2 (Figure 3A and Supplementary Tables S1, S3). *en1a*, *mhc1uba*, and *mhc2dab* were in the top 10 Down genes in both Y1 and Y2, while *capgb*, *c1qb*, and *entpd2b* were in the top 10 Up genes in Y1 (Figure 3B) and *isl1*, *msh4*, and *tfa* were in the top 11 Up genes in Y2 (Figure 3C). Eight hundred forty-six genes (335 Up and 511 Down) were shared between Y1 and Y2 and harbored many members of gene families encoding for solute carriers, calcium channels, ring finger proteins, and protocadherins (Figure 3A and Supplementary Table S3). The top 40 GO terms in Y1 and Y2 overlapped by 25% (Figures 3D,E). We also defined altered GO terms and Reactome pathways by comparing the Y1 and Y2 groups (Supplementary Figures S4, S5 and Supplementary Tables S4, S5). There were 15 KEGG pathways significantly enriched in Y1 (Figure 3F and Supplementary Table S5). The qPCR results confirmed that the regeneration-related DEGs *csf1ra* and *cd74b* (Chitu et al., 2016; Hwang et al., 2017) were upregulated in the lesioned hemisphere (Figure 3G) and *mpeg1.1* and *gfap* (Kroehne et al., 2011; Paredes et al., 2015) in both hemispheres (Figure 3H). Moreover, we observed a remarkable increase in the number of proliferating cells at the ventricular zone of the telencephalon in the lesioned hemisphere, detected by proliferating cell nuclear antigen (PCNA) antibody staining (Figures 3I,J). Thus, we conclude that the number of DEGs is higher in both hemispheres during the proliferative stage in comparison to the early wound healing stage and that 41% of all DEGs are shared between the lesioned and unlesioned hemispheres at this stage.

### The Early Wound Healing and Proliferative Stages of Brain Regeneration Are More Different Than Similar

**FIGURE 4:**
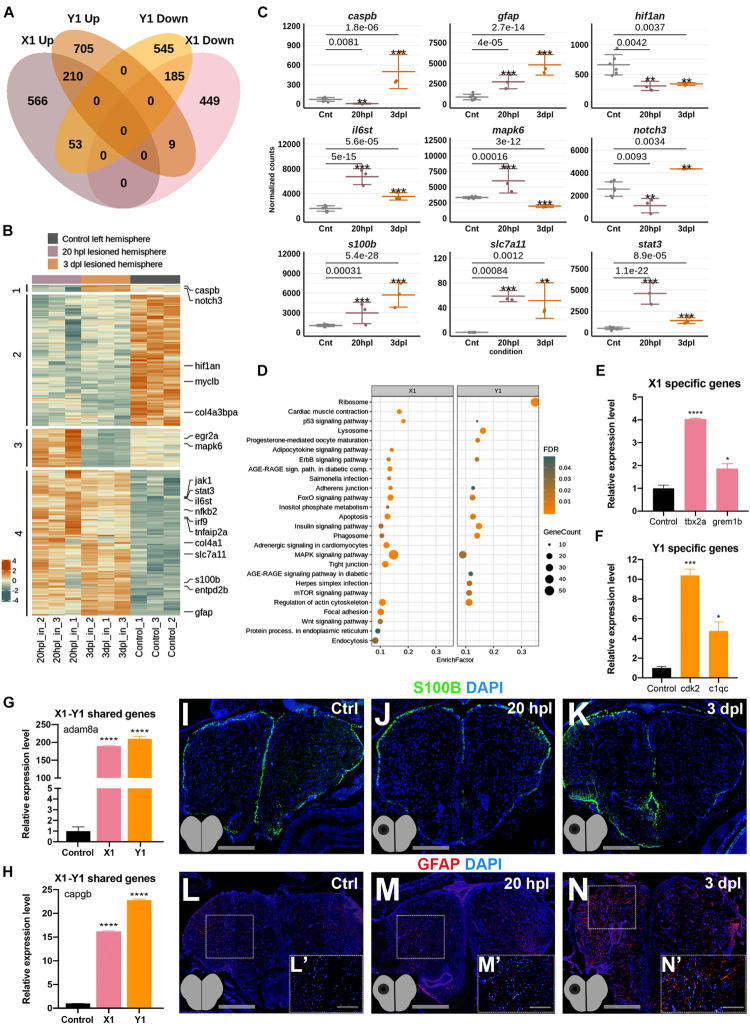
Early wound healing and proliferative stages of brain regeneration are more different than similar. **(A)** The Venn diagram shows the number of upregulated (*Up*) or downregulated (*Down*) differentially expressed genes (DEGs) in the lesioned 20-hpl (*X1*, *pink*) and lesioned 3-dpl (*Y1*, *orange*) hemispheres and the overlap between each set of DEGs. Out of 1,472 genes, 1,015 were significantly changed only in X1, while 1,250 out of 1,707 genes were so in Y1. There were 457 genes shared between X1 and Y1. *Darker* and *lighter shades* indicate upregulation and downregulation, respectively. **(B)** The heatmap shows the expressions of genes shared between X1 and Y1. Each *column* represents a single hemisphere from a single telencephalon and each *row* shows a single gene. The *scale bar* shows counts *Z*-scores from high to low expression, represented by a color gradient from *orange* to *green*, respectively. **(C)** Dot plot representation of normalized transformed read counts of a representative set of genes shared between X1 and Y1 (*gray*: control samples; *pink*: 20-hpl lesioned hemisphere; *orange*: 3 dpl lesioned hemisphere). The mean ± standard deviations (SD) of three independent experiments are plotted. **(D)** STRING bioinformatics tool was used to show the most significantly enriched KEGG pathways based on the transcriptional changes in X1 and X2. The *dot plot* represents KEGG pathways enriched in X1 and Y1 by using all significantly changed genes in these comparisons based on FDR and EnrichFactor. **(E,F)** Relative expression levels of DEGs that are unique to X1 (*tbx2a* and *grem1b*) or unique to Y1 (*cdk2* and *c1qc*). **(G,H)** Relative expression levels of DEGs shared between X1 and Y1 (*adam8a* and *capgb*). Statistical significance was evaluated using unpaired *t*-test. ****p < 0.0001. *Error bars* represent ± standard error of the mean (SEM, *n* = 3). **(I–K)** Anti-S100β staining of the control, 20 hpl, and 3 dpl brain sections. **(L–N,L′–N′)** Anti-GFAP staining of the control, 20 hpl, and 3 dpl brain sections with *boxed* areas magnified. Sections are counterstained for DAPI. *Scale bars*, 200 μm in **(L–N)**; 100 μm in **(L′–N′)**. *STRING*, Search Tool for the Retrieval of Interacting Genes/Proteins; *KEGG*, Kyoto Encyclopedia of Genes and Genomes; *FDR*, false discovery rate; *hpl*, hours post-lesion; *dpl*, days post-lesion. See section “Materials and Methods” for the definition of DEGs covered in X and Y comparisons. *p < 0.05, **p < 0.01, ***p < 0.001, and ****p < 0.0001.

Next, to compare the molecular and cellular events that occur in the lesioned hemisphere of the telencephalon at its early wound healing and proliferative stages, we compared the transcriptome profiles of the X1 and Y1 groups in detail. There were 1,015 DEGs (566 Up and 449 Down) that were unique to X1 and 1,250 DEGs (705 Up and 545 Down) were unique to Y1 (Figure 4A and Supplementary Table S6). We found that 457 genes (27% of X1 DEGs and 31% of Y1 DEGs) were shared between X1 and Y1 (Figure 4A and Supplementary Table S6). Among them, 395 genes were regulated in the same way, i.e., Up in both or Down in both, while 62 were regulated inversely. We next intersected the DEGs in X1 and Y1 to reveal genes differentially expressed at both time points. The heatmap displayed four groups of shared genes: group 1 with nine genes (e.g., *caspb* and *notch3*) Down in X1 and Up in Y1; group 2 with 185 genes (e.g., *hif1an*) Down in both X1 and Y1; group 3 with 53 genes (e.g., *mapk6*) Up in X1 and Down in Y1; and group 4 with 210 genes (e.g., *stat3*, *il6st*, *slc7a11*, and *s100b*) Up in both X1 and Y1 (Figures 4B,C and Supplementary Table S6). To compare the molecular data obtained from 20 hpl and 3 dpl using GO term enrichment, we used three groups of DEGs: (1) DEGs unique to X1; (2) DEGs unique to Y1; and (3) DEGs shared between X1 and Y1. GO term and KEGG pathway analyses showed enrichment of the Wnt signaling pathway exclusively in X1, whereas several other signaling pathways including p53, ErbB, FoxO, apoptosis, insulin, and MAPK were shared between X1 and Y1; mTOR signaling was uniquely enriched in Y1 (Figure 4D, Supplementary Figure S6, and Supplementary Table S5). To validate differential gene expression, we selected DEGs that are unique to X1 (*tbx2a* and *grem1b*), unique to Y1 (*cdk2* and *c1qc*), or shared between X1 and Y1 (*adam8a* and *capgb*), which are all known to be involved in tissue regeneration (Schlomann et al., 2000; Garnier et al., 2009; Satoh and Makanae, 2014; Peterson et al., 2015; Wang et al., 2015; Tang et al., 2017). All selected DEGs became upregulated after injury in the corresponding hemisphere, as detected by qPCR (Figures 4E–H). Furthermore, immunofluorescence staining for the glial cell markers S100β and glial fibrillary acidic protein (GFAP) confirmed upregulation at both 20 hpl and 3 dpl (Figures 4I–N). Overall, the early wound healing and proliferative stages of brain regeneration share less than a third of their individual pools of DEGs, strongly suggesting that the molecular and cellular mechanisms of regeneration substantially differ at these stages.

### Wnt/β-Catenin Signaling Is Activated During the Early Wound Healing Stage in the Regenerating Brain

**FIGURE 5:**
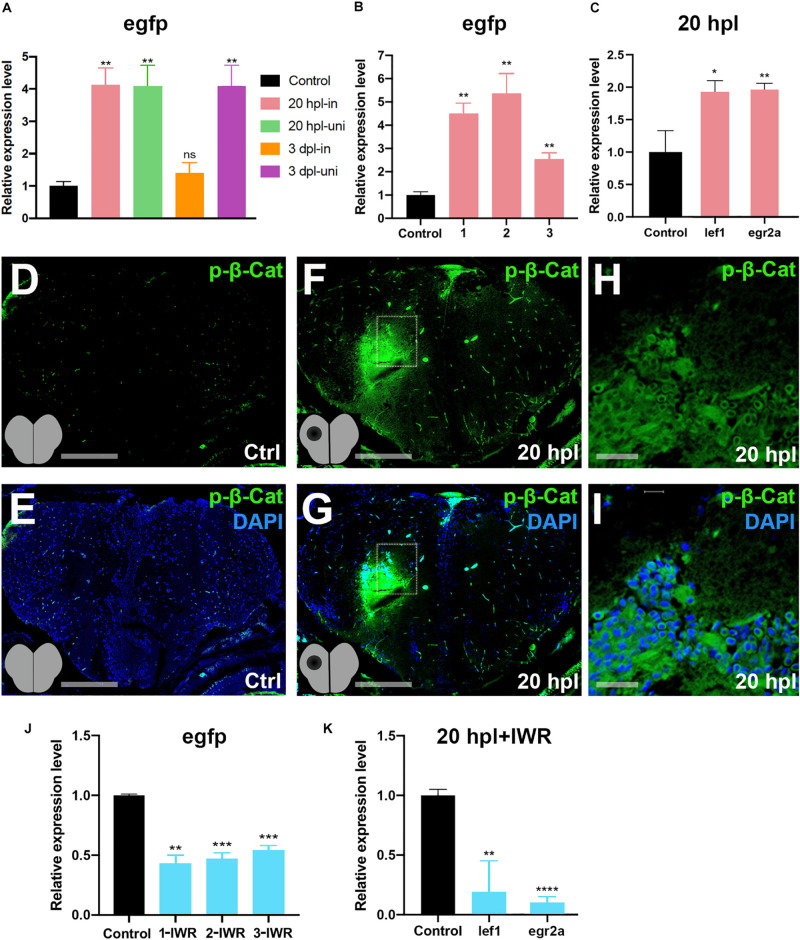
Wnt/β-catenin signaling is activated during the early wound healing stage in the regenerating brain. **(A)** Relative expression levels of *egfp* as a reporter of Wnt/β-catenin signaling activity in the lesioned and unlesioned hemispheres of the telencephalon during the early wound healing and proliferative stages of regeneration. Three hemispheres were pooled for each sample. **(B)** Relative expression levels of *EGFP* as a reporter of Wnt/β-catenin signaling activity in the lesioned hemispheres of three independent telencephalon samples used for RNA sequencing during the early wound healing stage. **(C)** Relative expression levels of the known Wnt target genes *lef1* and *egr2a* in the lesioned hemisphere of the telencephalon during the early wound healing stage. **(D,E)** Anti-phospho-β-catenin staining of control brain sections. **(F,G)** Anti-phospho-β-catenin staining of 20-hpl brain sections. The lesioned area is *boxed* and shown in **(H,I)**. Sections are counterstained for DAPI. *Scale bars*, 200 μm in **(D–G)**; 25 μm in **(H,I)**. **(J)** Relative expression levels of *egfp* as a reporter of Wnt/β-catenin signaling activity in lesioned + IWR-1-treated hemispheres of three independent telencephalon samples collected at 20 hpl. **(K)** Relative expression levels of the Wnt target genes *lef-1* and *egr2a* in the pooled three lesioned + IWR-1-treated hemispheres at 20 hpl. Statistical significance was evaluated using unpaired *t*-test. **p < 0.01 and ***p < 0.001. *Error bars* represent ± standard error of the mean (SEM, *n* = 3). *hpl*, hours post-lesion. *p < 0.05, **p < 0.01, ***p < 0.001, and ****p < 0.0001.

Comparative analyses of the GO terms and KEGG pathways for the genes that are differentially expressed at 20 hpl and 3 dpl have disclosed a strong and specific enrichment of gene sets related to Wnt signaling at 20 hpl: “Wnt signaling pathway,” “canonical Wnt signaling pathway,” “β-catenin destruction complex,” and “negative regulation of canonical Wnt signaling pathway” (Figure 4D, Supplementary Figure S6, and Supplementary Tables S4, S5). Thus, we next aimed to address the role and molecular targets of Wnt/β-catenin signaling, the so-called canonical Wnt pathway, during the early wound healing stage of brain regeneration. Wnt/β-catenin signaling is one of the most common intracellular signal transduction pathways activated in response to injury of virtually all tissues/organs (Ozhan and Weidinger, 2014). While Wnt signaling has been revealed to promote regeneration of the optic tectum, the spinal cord, and the tectum of the midbrain in zebrafish (Shimizu et al., 2012, 2018; Strand et al., 2016; Wehner et al., 2017; Lindsey et al., 2019), it has not yet been associated with the regeneration of the telencephalon. We have used the Tg(6XTCF:dGFP) transgenic zebrafish reporter of Tcf/Lef-mediated transcription, which has been shown to sensitively detect Wnt/β-catenin pathway activity in several cellular contexts (Shimizu et al., 2012). GFP expression in the reporter line is difficult to detect because of the unstable nature of GFP, leading to a low level of fluorescence in the adult zebrafish. We have performed immunofluorescence staining for anti-GFP at 20 hpl and detected Wnt activity in the lesioned area, most likely in the brain endothelial and blood cells (Supplementary Figure S7). Quantification of pathway activity by qPCR in the reporter line indeed showed an upregulation of Wnt signaling in both the lesioned and unlesioned hemispheres at 20 hpl (Figure 5A). Interestingly, signaling became downregulated in the lesioned hemisphere at 3 dpl while staying elevated in the unlesioned hemisphere, suggesting that it is specifically suppressed in the lesioned hemisphere after the wound healing stage. We also verified the significant activation of Wnt/β-catenin signaling at 20 hpl in the lesioned telencephalic hemispheres of the samples that were sent for RNA sequencing (Figure 5B). The Wnt/β-catenin target genes *lef1* and *egr2a* were likewise upregulated at 20 hpl in the lesioned hemispheres (Figure 5C). Immunofluorescence staining of brain sections showed an elevation of phospho-β-catenin (Ser675) levels in the lesioned hemisphere, especially in the vicinity of the lesion, indicating an increased transcriptional activity of β-catenin (Figures 5D–I). Considering the high levels of Wnt/β-catenin activity in the endothelial compartment of the zebrafish and mouse brains (Moro et al., 2012; Martowicz et al., 2019), it is likely that these Wnt-positive cells are non-CNS cells. To unravel how Wnt/β-catenin signaling regulates brain regeneration at the molecular level, we set out to identify its target genes in the early stage where signaling was significantly enhanced. To this purpose, we initially confirmed that Wnt/β-catenin signaling could be efficiently inhibited by the Wnt antagonist IWR-1, as evidenced by the significant reduction in the expressions of *egfp* in the Tg(6XTCF:dGFP) Wnt reporter and the canonical Wnt target genes *lef1* and *egr2a* in the lesioned hemisphere at 20 hpl (Figures 5J,K). Thus, we conclude that Wnt/β-catenin signaling is activated in the early wound healing stage of brain regeneration and returns to control level in the lesioned site at the early proliferative phase.

### Inhibition of Wnt/β-Catenin Signaling During the Early Wound Healing Stage Identifies 119 Target Genes That Are Positively Regulated by the Pathway

**FIGURE 6:**
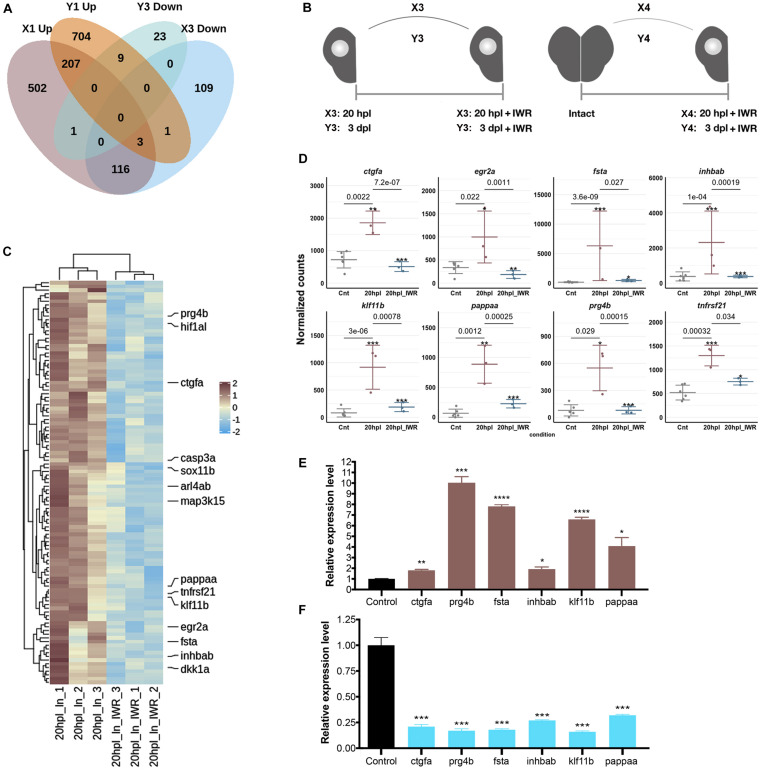
Inhibition of Wnt/β-catenin signaling during the early wound healing stage identifies 119 target genes that are positively regulated by the pathway. **(A)** The Venn diagram shows the number of differentially expressed genes (DEGs) that are upregulated (*Up*) in the lesioned 20-hpl (*X1*, *dark pink*), downregulated (*Down*) in the lesioned + IWR-1-treated 20-hpl (*X3*, *light blue*), Up in the lesioned 3-dpl (*Y1*, *dark orange*), or Down in the lesioned + IWR-1-treated 3-dpl (*Y3*, *turquoise*) hemispheres and the overlap between each set of DEGs. There were 119 genes Up in X1 and Down in X3, while only nine genes were Up in Y1 and Down in Y3. **(B)** Preparation of RNA samples from the lesioned hemisphere at 20 hpl or 3 dpl. *X3*: 20-hpl lesioned hemisphere after IWR treatment vs. 20-hpl lesioned hemisphere; *Y3*: 3-dpl lesioned hemisphere after IWR treatment vs. 3-dpl lesioned hemisphere; *X4*: 20-hpl lesioned hemisphere after IWR treatment vs. control; *Y4*: 3-dpl lesioned hemisphere after IWR treatment vs. control. **(C)** The heatmap shows the Wnt target genes that are Up in X1 and Down in X3. Each *column* represents a single hemisphere from a single telencephalon and each *row* shows a single gene. The *scale bar* shows counts *Z*-scores from high to low expression, represented by a color gradient from *orange* to *green*, respectively. **(D)** Dot plot representation of normalized transformed read counts of a representative set of Wnt target genes that are Up in X1 and Down in X3 (*brown*: 20-hpl lesioned hemispheres; *blue*: 20-hpl lesioned + IWR-1-treated hemispheres). The mean ± standard deviations (SD) of three independent experiments are plotted. **(E,F)** Relative expression levels of the Wnt target genes that are Up in X1 and Down in X3 (*ctgfa*, *prg4b*, *fsta*, *inhbab*, *klf11b*, and *pappaa*). Statistical significance was evaluated using unpaired *t*-test. *p < 0.05, **p < 0.01, ***p < 0.001, and ****p < 0.0001. *Error bars* represent ± standard error of the mean (SEM, *n* = 3). See section “Materials and Methods” for the definition of DEGs covered in X and Y comparisons.

Next, to identify the Wnt targetome at the early wound healing and proliferative stages of brain regeneration, we suppressed Wnt/β-catenin signaling starting from the time of injury to 20 hpl or to 3 dpl by treating the zebrafish with the Wnt antagonist IWR-1 and had the transcriptome of the lesioned hemispheres sequenced at 20 hpl or 3 dpl. There were 293 genes (64 Up and 229 Down) in X3 and 103 genes (70 Up and 33 Down) in Y3 comparisons (Figure 6A and Supplementary Table S1). We termed DEGs that are Up in X1 and Down in X3 as the positively regulated Wnt targetome at 20 hpl and the genes that are Up in Y1 and Down in Y3 as the positively regulated Wnt targetome at 3 dpl (Figures 1A, 6B). The Wnt targetome that was composed of 119 genes at 20 hpl (Figures 6C,D and Supplementary Table S7) sharply narrowed down to nine genes at 3 dpl (Supplementary Figure S9 and Supplementary Table S7). Next, to validate the Wnt targetome at 20 hpl, we measured the expression levels of some genes selected from the targetome by qPCR. All selected genes were significantly upregulated in the lesioned hemisphere following injury and became downregulated when Wnt signaling was inhibited by IWR-1 from the time of injury until 20 hpl (Figures 6E,F). These results strongly suggest a key role for Wnt/β-catenin signaling in the early wound healing stage of regeneration.

### Inhibition of Wnt/β-Catenin Signaling During the Early Wound Stage Results in a Marked Alteration of the Gene Expression Profiles Represented in KEGG Pathway Enrichment

**FIGURE 7:**
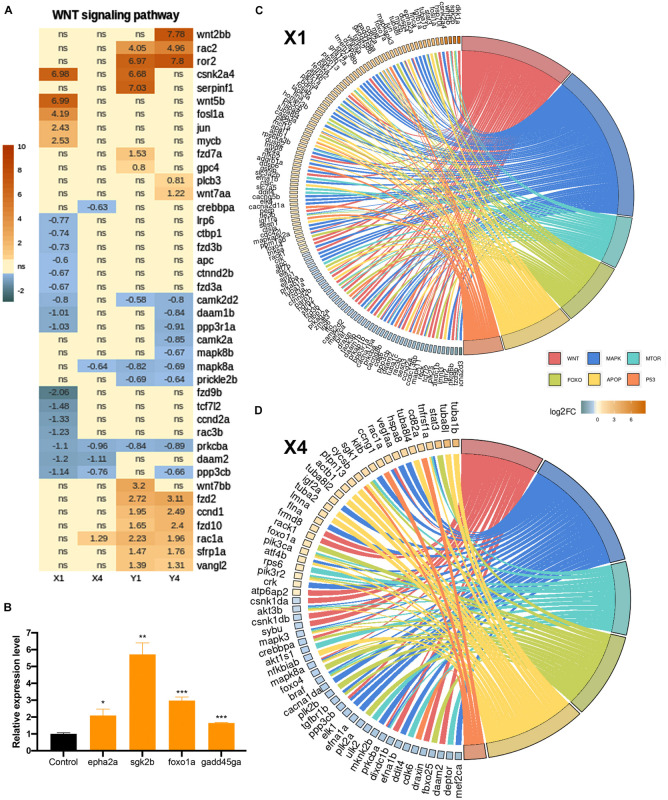
Inhibition of Wnt/β-catenin signaling during the early wound stage results in a marked alteration of the gene expression profiles represented in Kyoto Encyclopedia of Genes and Genomes (KEGG) pathway enrichment. Heatmap representation of the uniquely changed genes in **(A)** the Wnt signaling pathway in X1, X4, Y1, and Y4 comparisons. Each *column* represents a comparison according to the DESeq2 results and each *row* shows a single gene. The *scale bar* shows log_2_ of the fold change values (when statistically significant) from high to low fold change, represented by a color gradient from *orange* to *blue*, respectively. Statistical significance was evaluated using Wald test. *ns*, non-significant. **(B,C)** GOChord plot of selected GO terms belonging to the biological process (BP) sub-ontology and KEGG pathways for X1 and X4 comparisons, respectively. The genes related to the Wnt-KEGG pathway and Wnt-Gene Ontology BP are intersected and represented as one group. The genes are linked to their assigned pathways by *ribbons* and ordered according to their log_2_ of the fold change values from high to low fold change, represented by a color gradient from *orange* to *blue*, respectively. **(D)** Relative expression levels of pathway-related genes (*epha2a*, *sgk2b*, *foxo1a*, and *gadd45ga*). Statistical significance was evaluated using unpaired *t*-test. *p < 0.05, **p < 0.01, and ***p < 0.001. *Error bars* represent ± standard error of the mean (SEM, *n* = 3). See section “Materials and Methods” for the definition of DEGs covered in X and Y comparisons.

Among the KEGG pathways that were significantly enriched in the lesioned hemisphere at 20 hpl (X1) or 3 dpl (Y1), several of them, including the p53 signaling pathway, apoptosis, MAPK signaling pathway, and FoxO signaling pathway, were shared between X1 and Y1, whereas the Wnt and mTOR signaling pathways were unique to X1 and Y1, respectively (Figures 2F, 3F, 4D). Thus, we set out to compare how these KEGG pathways are altered in the lesioned hemisphere at 20 hpl and 3 dpl in response to inhibition of Wnt/β-catenin signaling. We termed the comparison of the 20 hpl lesioned hemisphere + IWR treatment to control as X4 and the comparison of the 3 dpl lesioned hemisphere + IWR treatment to control as Y4 (Figure 6B). There were 868 genes (387 Up and 481 Down) differentially expressed in X4 and 1,977 (1,179 Up and 798 Down) were so in Y4 (Supplementary Table S1). The genes we obtained from the KEGG and AmiGO databases included substantial numbers of genes involved in the Wnt, MAPK, apoptosis, mTOR, p53, and FoxO signaling pathways (Supplementary Table S8). To compare the alterations in the gene expression profiles related with these pathways during two regenerative stages, we intersected these genes with DEGs in X1, X4, Y1, and Y4. Strikingly, the number of significantly altered genes in X1 decreased dramatically after IWR treatment in X4 for all pathways (Figures 7A–D and Supplementary Figure S10 compare the total number of brown and blue shaded boxes in X1 and X4; Supplementary Table S8). Among the Wnt signaling pathway genes that were detected in the two KEGG pathways, “WNT signaling pathway” (Figure 7A) and “WNT_GO signaling pathway” (Supplementary Figure S10A), 12 of the 14 genes that were Up in X1 (*csnk2a4*, *wnt5b*, *fosl1a*, *jun*, *mycb*, *dkk1a*, *gskip*, *ppm1ab*, *tnksa*, *fermt2*, *tmem198b*, and *tle3b*) did not significantly change any more after IWR treatment in X4, indicating that these 12 genes are positively regulated Wnt targets (Figure 7A and Supplementary Figure S10A compare the number of brown shaded boxes in X1 and X4). 18 of the 24 genes that were Down in X1 (*lrp6*, *ctbp1*, *fzd3b*, *apc*, *ctnnd2b*, *fzd3a*, *camk2d2*, *daam1b*, *ppp3r1a*, *fzd9b*, *tcf7l2*, *ccnd2a*, *rac3b*, *ndrg2*, *fto*, *reck*, *ankrd6b*, and *ccdc136b*) did not change any more in X4, suggesting that these 18 genes are negatively regulated Wnt targets (Figure 7A and Supplementary Figure S10A compare the number of blue shaded boxes in X1 and X4). On the contrary, the number of altered genes in Y1 did not change much either in Y4 (Figure 7A and Supplementary Figure S10 compare the total number of brown and blue shaded boxes in Y1 and Y4; Supplementary Table S8). Accordingly, 20 out of 30 DEGs determined in the Wnt pathway-related genes that were differentially regulated in Y1 were likewise regulated after IWR treatment in Y4 (Figure 7A and Supplementary Figure S10A). Finally, we were able to validate some selected genes, i.e., *epha2a* as a MAPK pathway-related gene, *sgk2b* and *foxo1a* as FoxO signaling-related genes, and *gadd45ga* as a p53 pathway-related gene at 20 hpl (Figure 7B). Taken together, these data indicate that Wnt/β-catenin signaling controls the expression of a wide range of genes related to various signaling pathways, including p53, apoptosis, FoxO, MAPK, and mTOR, at the early wound healing stage of brain regeneration.

## Discussion

Although the zebrafish telencephalon has been carefully explored for its neurogenic niches that harbor stem or progenitor cells expressing different marker genes, comparative analysis of the transcriptome profiles at the early stages of telencephalon regeneration has not been performed yet. This is the first study that uncovers the global gene expression profiles of the regenerating zebrafish telencephalon in the lesioned and unlesioned hemispheres at two early stages of regeneration, which we defined as 20 hpl, corresponding to the early wound healing stage, and 3 dpl, to the early proliferative stage. Using comparative gene expression profiling, we describe for the first time that: (i) the total number of DEGs in the lesioned hemisphere is substantially higher than that in the unlesioned one at 20 hpl; (ii) the number of DEGs in 3 dpl is elevated as compared to 20 hpl and that 40% of all DEGs at 3 dpl are shared between the lesioned and unlesioned hemispheres; (iii) transcriptomes at 20 hpl and 3 dpl share less than a third of their DEGs; (iv) Wnt/β-catenin signaling is activated at 20 hpl, controlling the transcription of a large pool of target genes, 119 of which are positively regulated by the pathway; and (v) Wnt/β-catenin signaling results in a marked alteration of the gene expression profiles represented as enrichment of the KEGG pathways, including p53, apoptosis, MAPK, mTOR, and FoxO, at the early wound healing stage of brain regeneration.

Injury-induced proliferative response has been proposed to be primarily confined to the lesioned hemisphere of the telencephalon (Marz et al., 2011). Nevertheless, several genes are upregulated both in the lesioned and unlesioned hemispheres, though not to the same degree (Kroehne et al., 2011; Kizil et al., 2012b). The difference in the responses of the two hemispheres to injury could be due to differences in the methods used to induce a telencephalic injury. Nevertheless, whether the unlesioned part could be used as an effective internal control is a matter of debate. Strikingly, studies of hemispherectomy, where one cerebral hemisphere is completely removed or functionally disconnected, have demonstrated that the contralesional hemisphere is able to undertake various functions of the lesioned side, most likely due to a strong interaction between the lesioned and the intact halves in brain injuries (Sebastianelli et al., 2017). Such an interplay would be inevitably reflected as differential gene expression after injury not only in the lesioned but also in the unlesioned site of the telencephalon. Our results show that, at both 20 hpl and 3 dpl, many DEGs were shared between the lesioned (X1 or Y1) and the unlesioned (X2 or Y2) hemispheres and that the number of shared DEGs at 3 dpl was far higher than that at 20 hpl (see Figures 2A, 3A). Thus, the lesioned and unlesioned hemispheres indeed appear to interact during telencephalon regeneration, and the level of this interaction increases from the early wound healing to the proliferative stage. The injury response appears to be most intense and peculiar at the wound site during the early wound healing stage and extends outside as the proliferative stage starts, leading to a considerable difference between the transcriptional programs activated at these two stages.

Cytokines released from innate immune cells play key roles in the regulation of the acute inflammatory response that is required for functional regeneration of the zebrafish CNS after injury (Kyritsis et al., 2012; Elsaeidi et al., 2014; Fuller-Carter et al., 2015; Tsarouchas et al., 2018). Our data showed prominent upregulation of the anti-inflammatory cytokine genes *il11a*, *il21*, and *il6st*, cytokine receptor encoding *il17ra1a*, the tumor necrosis factor receptor superfamily (TNFRSF) genes *tnfrsf18*, *tnfrsf21*, and the genes related to cytokine response *junba*, *jun*, *rel*, and *timp2a* at 20 hpl, suggesting that signaling through these early response cytokines is essential to orchestrate innate immune response (Opal and DePalo, 2000; Placke et al., 2010). Apoptosis is also activated in the early regenerative phase of various tissues including *Xenopus* tail, mouse liver, and cornea and is indispensable for effective wound healing (Tseng et al., 2007; Wilson et al., 2007; Li et al., 2010). Remarkably, we observed a significant upregulation of the apoptosis-related genes including *apaf1*, *baxa*, *traf4b*, *tgfb2*, *sgk1*, and *casp3a* selectively in the lesioned hemisphere at 20 hpl (Elmore, 2007; Gilbert et al., 2016; Liu et al., 2017; Kreckel et al., 2019). Strikingly, many of them were downregulated when Wnt/β-catenin signaling was suppressed, pointing out a positive regulatory role for Wnt signaling in apoptosis during early brain regeneration. Dual-specific phosphatase (DUSP) genes were also upregulated in the lesioned hemisphere. Among these, *dusp6* has been shown to promote cell death in different contexts (Domercq et al., 2011; Piya et al., 2012). Apoptosis has been proposed as a key factor to resolve inflammation by driving the conversion of immune response into a wound healing response in the early phases of tissue repair (Brown et al., 1997; Wu and Chen, 2014). Thus, parallel elevations in the expression levels of genes related to cytokine signaling and apoptosis at 20 hpl could underlie the capacity of zebrafish telencephalon to convert the early inflammatory response into a healing ability and consolidate this time point as a representative of the early wound healing stage during telencephalon regeneration.

Angiogenic sprouting into the injured area has been described as another prominent event that takes place as early as 15 h after injury during heart regeneration in zebrafish (Marin-Juez et al., 2016). Significantly upregulated *vascular endothelial growth factor a-a* (*vegfaa*), a key regulator of angiogenesis, is essential for cardiomyocyte proliferation and heart regeneration (Marin-Juez et al., 2016). Likewise, the neuropilin genes *nrp1a*, *nrp1b*, and *nrp2a*, which encode for transmembrane receptors of vascular endothelial growth factors (VEGFs), are upregulated in injured hearts at 1 dpl and the glucose transporter gene *slc2a1b* upregulated in vascular endothelial cells in response to hypoxia (Delcourt et al., 2015; Guo and Vander Kooi, 2015; Lowe et al., 2019). CNS regeneration also involves an intense crosstalk between neurons and vascular niches that mediates angiogenesis mainly through the activation of VEGF and contributes to neuroprotection (Ruiz de Almodovar et al., 2009). We noted a significant upregulation of *vegfaa*, *nrp1a*, *nrp1b*, and *slc2a1b* genes specifically in the lesioned hemisphere at 20 hpl, arguing that injury activates a fast angiogenic sprouting mechanism during telencephalon regeneration.

Optic nerve injury in adult zebrafish induces the expression of the axon growth inhibitor suppressor of cytokine signaling 3 (*socs3*) in retinal ganglion cells, ultimately attenuating regeneration (Smith et al., 2009; Elsaeidi et al., 2014). We found that while upregulated at 20 hpl, *socs3a* was strongly downregulated at 3 dpl. *socs3b*, *sox11b*, and *klf6a*, which are associated with optic nerve regeneration (Veldman et al., 2007), were likewise induced at 20 hpl, with *sox11b* becoming downregulated at 3 dpl. More axon guidance genes including *robo1*, *tnc*, and *boc* (Lee et al., 2001; Connor et al., 2005; Schweitzer et al., 2005) were downregulated primarily in the lesioned hemisphere at 20 hpl. Such biphasic expression of axon growth inhibitors during brain regeneration suggests a stage-specific regulation of axon growth.

The proliferative response of the injured zebrafish telencephalon is driven by the RGCs and reaches a peak at the lesioned site around 3 dpl (Marz et al., 2011; Kizil et al., 2012c). At this stage, we observed that some quiescent RGC genes such as *fabp7a*, *mt-atp8*, *hipk1a*, *s100b*, and *mgll* were upregulated in both hemispheres, while *ptn*, *luzp2*, *psap*, *hepacama*, and *anxa11b* were only upregulated in the lesioned side (Kaslin et al., 2017; Lange et al., 2020). On the other hand, proliferating RGC genes including *tmsb4x*, *hmgb2a*, *hmga1a*, *rps23*, and *ran* were exclusively upregulated in the lesioned half, whereas the general proliferation marker *ccnd1* was upregulated in both halves (Lange et al., 2020). Besides, we found that many cell cycle-associated genes such as *pcna*, *mki67*, *rpl35*, *mdka*, *ccna2*, and *cdk1* were upregulated predominantly in the lesioned hemisphere (Sobecki et al., 2017). Together, these results validate 3 dpl as a highly proliferative phase dominated by RGC activities and reveal that this proliferative response is clearly more pronounced in the lesioned hemisphere.

As the resident macrophages of the CNS, microglia have been shown to engulf dead cells after brain injury in zebrafish (Herzog et al., 2019). Previous studies have identified various microglia- and/or macrophage-specific markers that are differentially regulated during regeneration of the retina and olfactory bulb (Oosterhof et al., 2017; Mitchell et al., 2019). At 3 dpl, a considerable number of microglial genes including *mhc1zba*, *marco*, *spi1b*, *csf1ra*, and *mfap4* were upregulated exclusively in the lesioned hemisphere, while a few including *mpeg1.1* and *sall3a* were upregulated in both hemispheres. This prominent emanation of microglial signature at 3 dpl correlates with the extensive proliferation of microglia induced by neuronal cell death (Oosterhof et al., 2017). Furthermore, we found 18 innate immune response genes (see Supplementary Table S4) to be upregulated at 3 dpl. Among them, *nfkb2* and *csf1ra* are involved in the activation of innate immune response during heart and tail fin regeneration (Petrie et al., 2014; Karra et al., 2015; Morales and Allende, 2019). Activation of the immune response thus needs to be analyzed together with the related signaling pathways to understand the mechanisms of proper tissue healing.

Wnt/β-catenin signaling appears to play a positive, regeneration-promoting role in most systems, from invertebrates that display whole-body regeneration to organs that mammals can completely or partially regenerate, such as the liver, skeletal muscle, and kidney (Whyte et al., 2012; Ozhan and Weidinger, 2014). Wnt signaling is also essential for the organs that regenerate completely only in the lower vertebrates, such as the appendages (Stoick-Cooper et al., 2007; Singh et al., 2018) and the eye (Hayashi et al., 2008; Patel et al., 2017). In the adult zebrafish, Wnt/β-catenin signaling is involved in the proliferation and differentiation of neural stem or progenitor cells in the hypothalamus, optic tectum, and spinal cord (Wang et al., 2009; Shi et al., 2015; Shitasako et al., 2017; Wehner et al., 2017). Our results further support the role of canonical Wnt signaling in regeneration by providing the first evidence of active signaling in the zebrafish telencephalon at a very early stage of regeneration, i.e., 20 hpl. Wnt/β-catenin signaling activity has been identified around the necrotic lesions in mouse liver and hair follicle regeneration models (Ito et al., 2007; Whyte et al., 2012; Zhao et al., 2019). Likewise, we have demonstrated the early activation of Wnt/β-catenin signaling near the lesioned area of the telencephalon. At 1 dpl, the telencephalic lesion is known to be filled with blood cells and is surrounded by macrophages/microglial cells, indicating an early infiltration of these cells into the site of tissue damage (Ayari et al., 2010). Therefore, identification of the types of blood and immune cells with active Wnt/β-catenin signaling at 20 hpl would unravel its role in the early wound healing process of brain regeneration. Interestingly, signaling becomes downregulated to control levels in the lesioned hemisphere at 3 dpl. Wnt/β-catenin signaling has been reported to have biphasic roles during heart, muscle, and liver development, where early and late signaling activities have diverse roles (Monga et al., 2001; Naito et al., 2006; Klaus et al., 2007; Ueno et al., 2007; Brack et al., 2008; Gessert and Kuhl, 2010). Thus, it is likely that Wnt signaling is regulated at least in a biphasic manner in the course of adult brain response to injury.

Several of the Wnt target genes we identified, including *sox11b*, *casp3a*, *klf11*, and *egr2a*, are known to be involved in the regulation of Wnt/β-catenin activity (Spittau and Krieglstein, 2012; Zaman et al., 2012; Zimmerman et al., 2013; Liu et al., 2019). Moreover, a wide range of genes related to the p53, apoptosis, MAPK, mTOR, and FoxO pathways are regulated by Wnt/β-catenin signaling during early wound healing, but become far less responsive to Wnt later at the proliferative stage. Wnt/β-catenin signaling has been implicated in the regulation of proliferation and differentiation of various progenitor cells during tissue regeneration *via* controlling the p53, mTOR, and FoxO pathways (Hirose et al., 2014; Peng et al., 2014; Maiese, 2015; Lund-Ricard et al., 2020). Besides, an intense crosstalk between the MAPK and Wnt signaling pathways has been deciphered in development, cancer, and regeneration (Caverzasio and Manen, 2007; Bikkavilli et al., 2008; Zhang et al., 2014). Our results reveal for the first time that these signaling pathways are activated in response to canonical Wnt pathway early after the telencephalon injury. Further functional studies will clarify how Wnt signaling interacts with these pathways to regulate the early regenerative response in brain regeneration.

In conclusion, our comparative transcriptome analyses of the regenerating zebrafish telencephalon at the early wound healing and proliferative stages reveal differentially expressed genes and altered biological pathways that control the cellular and molecular mechanisms of regeneration. The great burden caused by traumatic brain injuries and neurodegenerative diseases to humanity necessitates the development of therapeutic interventions, which is tightly dependent on unraveling these mechanisms that render possible effective brain regeneration.

## Data Availability Statement

All datasets have been deposited in ArrayExpress under the link: https://www.ebi.ac.uk/arrayexpress/experiments/E-MTAB-9321/ with the accession number “E-MTAB-9321”.

## Ethics Statement

The animal study was reviewed and approved by the Animal Experiments Local Ethics Committee of İzmir Biomedicine and Genome Center (IBG AELEC/IBG-HADYEK).

## Author Contributions

GO, YD, and GC designed the experiments. GC and YP performed the molecular and cell biology experiments. YD, SM, GH, and IP conducted the bioinformatics analyses. GO, YD, and GC wrote the manuscript. All authors contributed to the discussion.

## Conflict of Interest

The authors declare that the research was conducted in the absence of any commercial or financial relationships that could be construed as a potential conflict of interest.
