# Supplementary material for: Comparative Transcriptome Analysis of the Regenerating Zebrafish Telencephalon Unravels a Resource With Key Pathways During Two Early Stages and Activation of Wnt/β-Catenin Signaling at the Early Wound Healing Stage
Source: Front Cell Dev Biol. 2020 Oct 9;8:584604. doi: 10.3389/fcell.2020.584604 (PMC7581945; doi:10.3389/fcell.2020.584604)
Supplement: Supplementary file 9 [file Data_Sheet_1.pdf]

Supplementary Text for Demirci, Cucun et al.

Supplementary Figures

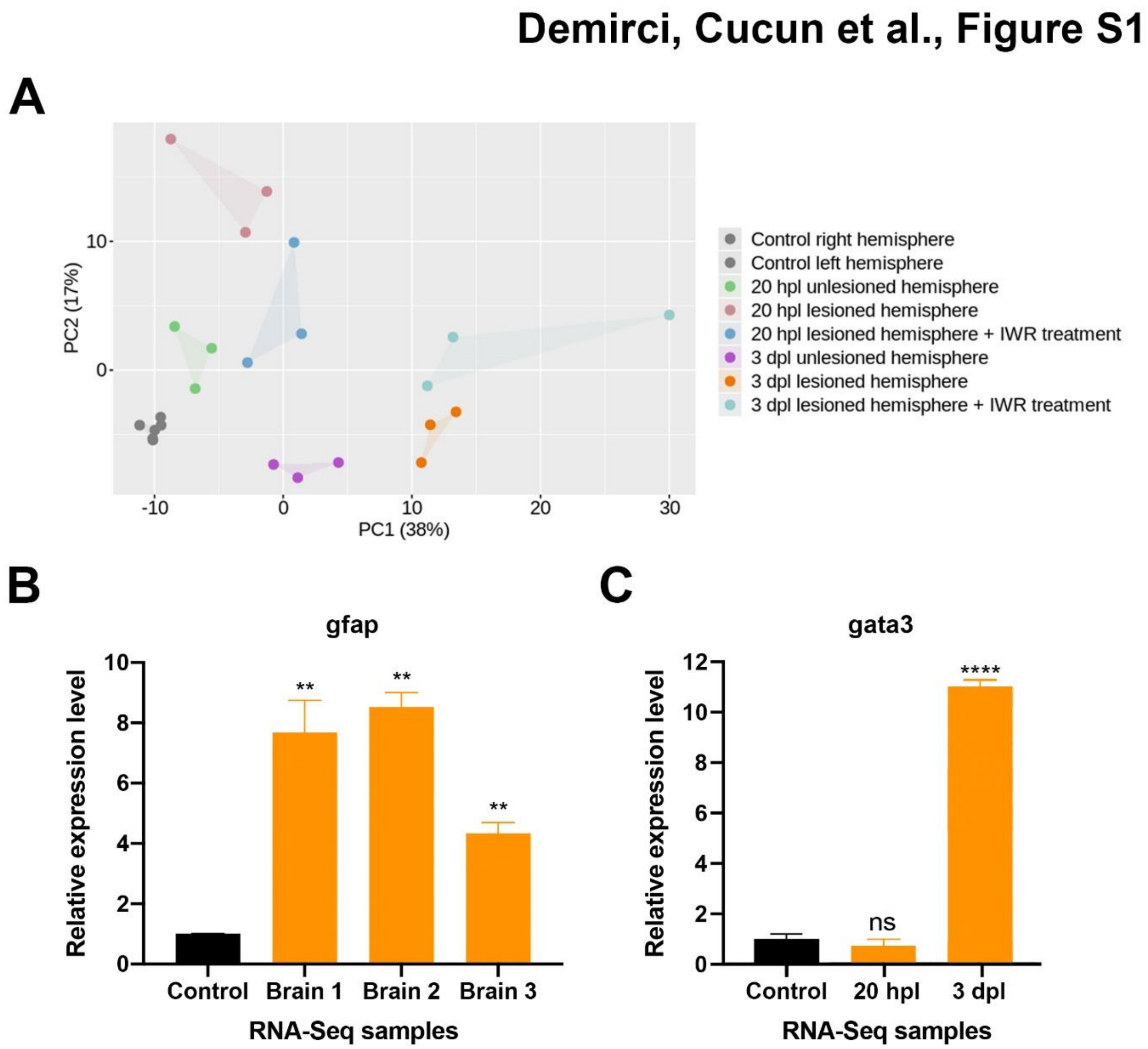

Supplementary Figure 1. Quantitative and qualitative control of RNA samples before and after RNA-seq (A) Principal component analysis (PCA) of all RNA-seq data. The PCA was conducted and plotted using package of “ggplot2”. Different colors of circle dots represents the eight groups of samples. Three circle dots with the same color refer to the three replicates of a sample group. Eight sample groups were well separated. Principal Component 1 (PC1, x-axis) represents 38% and PC2 (y-axis) represents 17% of total variation in the data. Relative expression levels of (B) the glia marker gene *gfap* in lesioned hemispheres of three independent telencephalon samples used for RNA-sequencing at 3 dpl and (C) injury-activated gene *gata3* in the pooled three lesioned hemispheres before injury and at 20 hpl and 3 dpl. Statistical significance was evaluated using

unpaired t-test. **p<0.01 and ***p<0.001. Error bars represent ± standard error of mean (SEM, n=3). hpl: hours post-lesion, dpl: days post-lesion, gfap: glial fibrillary acidic protein.

Demirci, Cucun et al., Figure S2

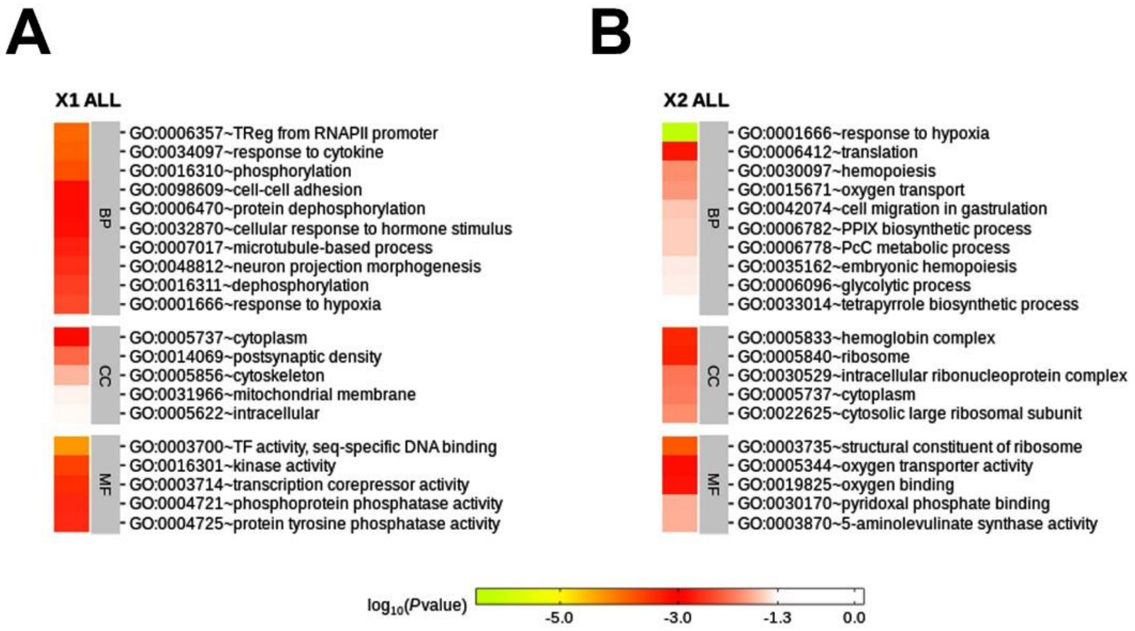

Supplementary Figure 2. GO terms altered in the lesioned and unlesioned hemispheres at the early wound healing stage of brain regeneration by using all significantly changed genes DAVID bioinformatics tool was used to show the most significantly enriched GO terms based on the transcriptional changes in (A) X1 and (B) X2 comparisons. All DEGs (X1 1472, X2 211) were used. The heatmap scale shows the $\log_{10}$ of the EASE P value for the most significantly enriched GO terms. DAVID: Database for Annotation, Visualization and Integrated Discovery, GO: Gene Ontology, BP: Biological Process, CC: Cellular Component, MF: Molecular Function. See Materials and Methods for definition of DEGs covered in X1 and X2.

Demirci, Cucun et al., Figure S3

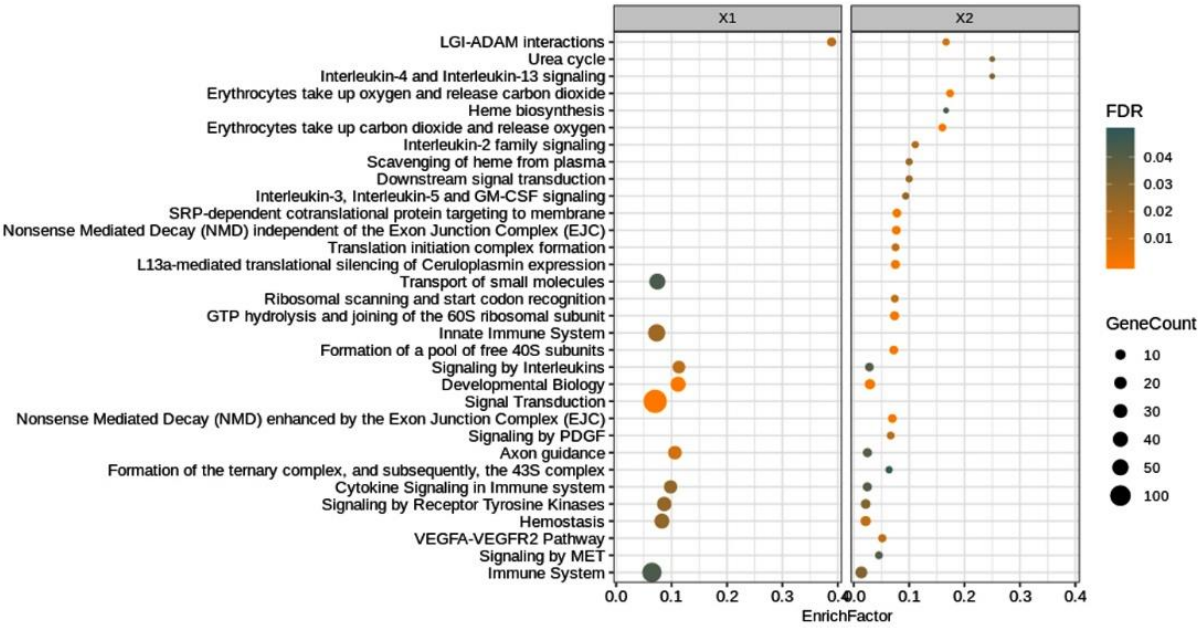

Supplementary Figure 3. Reactome pathways altered in lesioned and unlesioned hemispheres at the early wound healing stage of brain regeneration by using all significantly changed genes STRING bioinformatics tool was used to show the most significantly enriched Reactome pathways based on the transcriptional changes in X1 and X2. Dot plot represents Reactome pathways enriched in X1 and X2 by using all significantly changed genes (X1 1472, X2 211) in these comparisons based on FDR and EnrichFactor. STRING: Search Tool for the Retrieval of Interacting Genes/Proteins, FDR: false discovery rate. See Materials and Methods for definition of DEGs covered in X1 and X2.

Demirci, Cucun et al., Figure S4

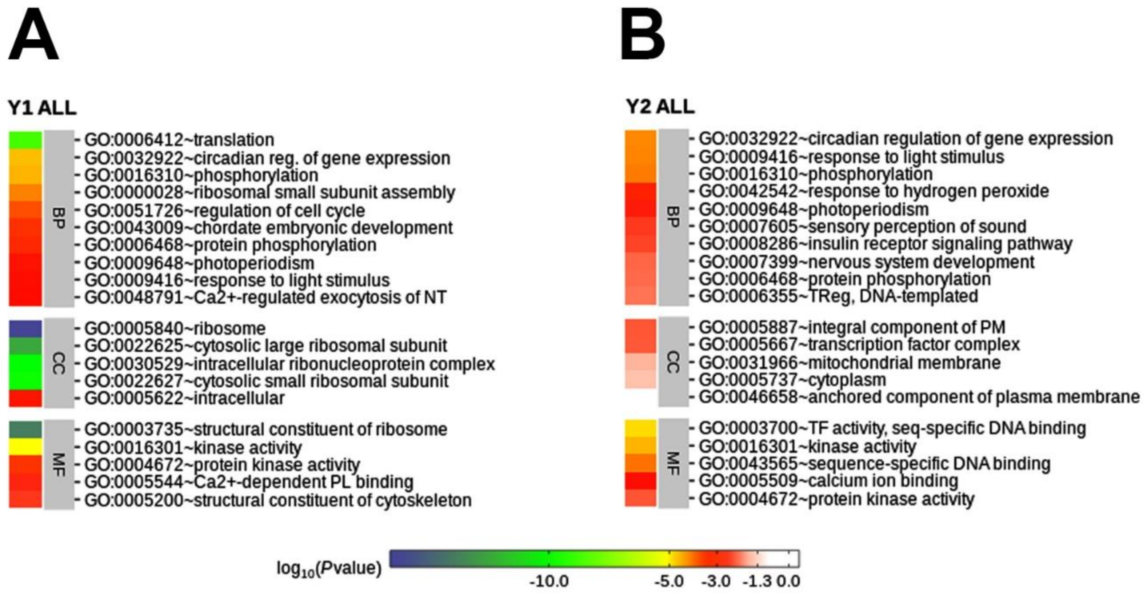

Supplementary Figure 4. GO terms altered in the lesioned and unlesioned hemispheres at the proliferative stage of brain regeneration by using all significantly changed genes DAVID bioinformatics tool was used to show the most significantly enriched GO terms based on the transcriptional changes in (A) Y1 and (B) Y2 comparisons. All DEGs (Y1 1707, X2 1215) were used. The heatmap scale shows the $\log_{10}$ of the EASE P value for the most significantly enriched GO terms. DAVID: Database for Annotation, Visualization and Integrated Discovery, GO: Gene Ontology, BP: Biological Process, CC: Cellular Component, MF: Molecular Function. See Materials and Methods for definition of DEGs covered in Y1 and Y2.

Demirci, Cucun et al., Figure S5

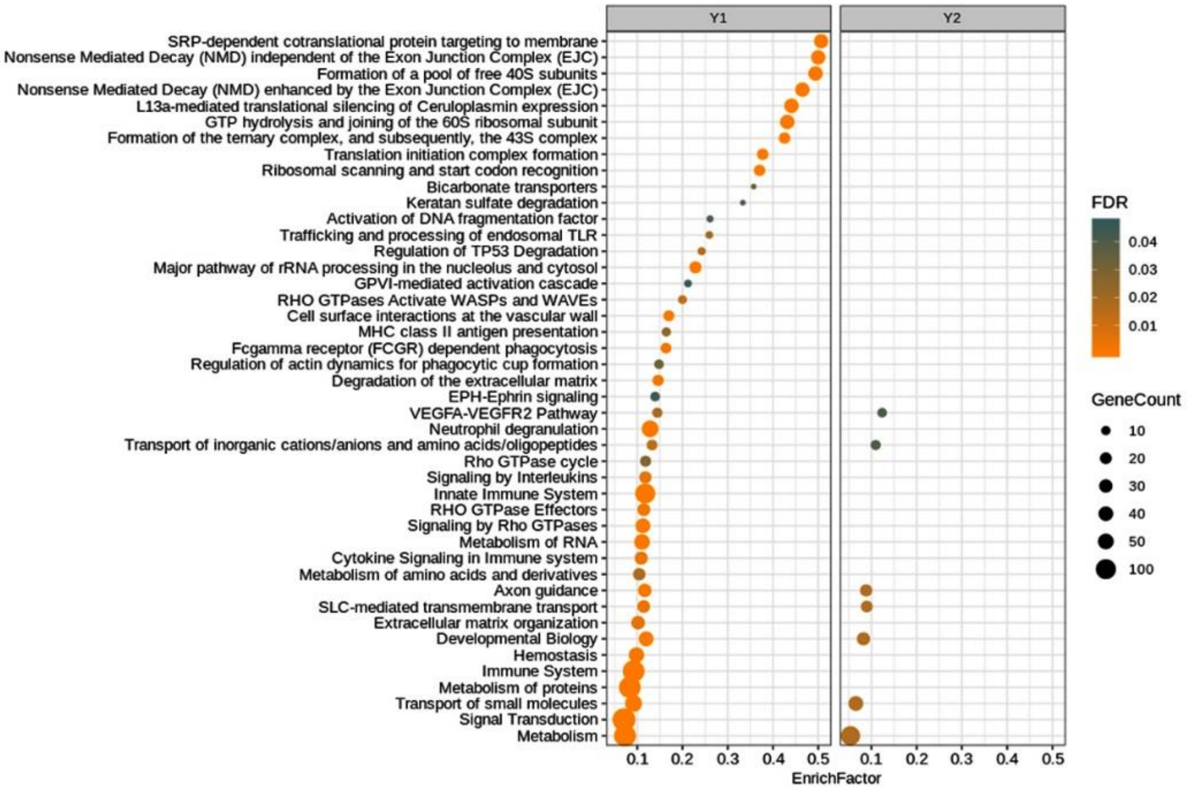

Supplementary Figure 5. Reactome pathways altered in lesioned and unlesioned hemispheres at the proliferative stage of brain regeneration by using all significantly changed genes STRING bioinformatics tool was used to show the most significantly enriched Reactome pathways based on the transcriptional changes in Y1 and Y2. Dot plot represents Reactome pathways enriched in Y1 and Y2 by using all significantly changed genes (Y1 1707, Y2 1215) in these comparisons based on FDR and EnrichFactor. STRING: Search Tool for the Retrieval of Interacting Genes/Proteins, FDR: false discovery rate. See Materials and Methods for definition of DEGs covered in Y1 and Y2 comparisons.

Demirci, Cucun et al., Figure S6

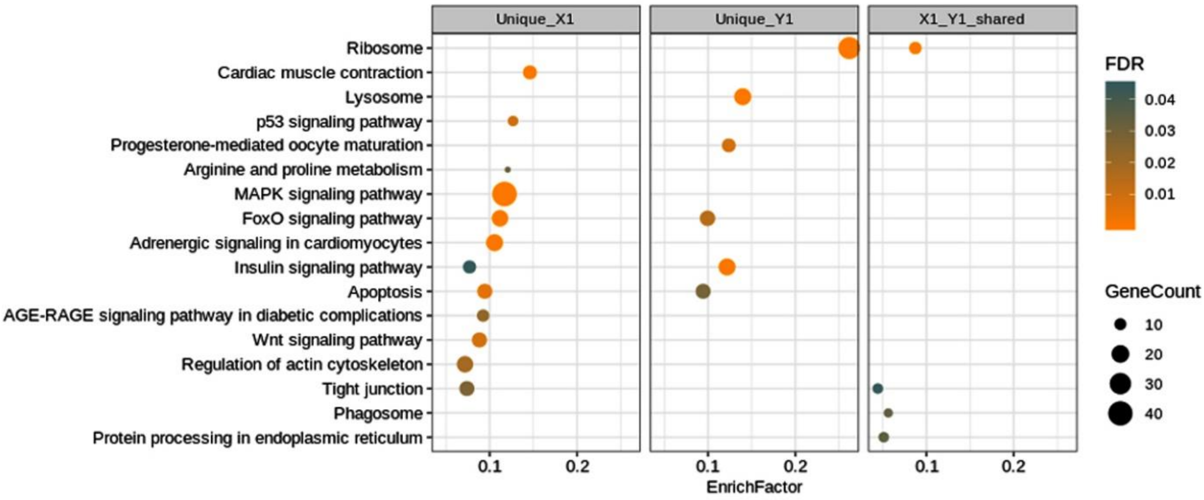

Supplementary Figure 6. KEGG pathways altered in lesioned and unlesioned hemispheres at the early wound healing and proliferative stages of brain regeneration by using shared and uniquely expressed genes STRING bioinformatics tool was used to show the most significantly enriched KEGG pathways based on the transcriptional changes in (A) genes unique to X1 and (B) genes unique to Y1 and (C) genes shared between X1 and Y1. 1015, 1250 and 457 DEGs were used for X1, Y1 and X1&Y1, respectively. Dot plot represents KEGG pathways enriched in X1 and Y1 comparisons based on FDR and EnrichFactor. STRING: Search Tool for the Retrieval of Interacting Genes/Proteins, KEGG: Kyoto Encyclopedia of Genes and Genomes, FDR: false discovery rate. See Materials and Methods for definition of DEGs covered in X and Y comparisons.

Demirci, Cucun et al., Figure S7

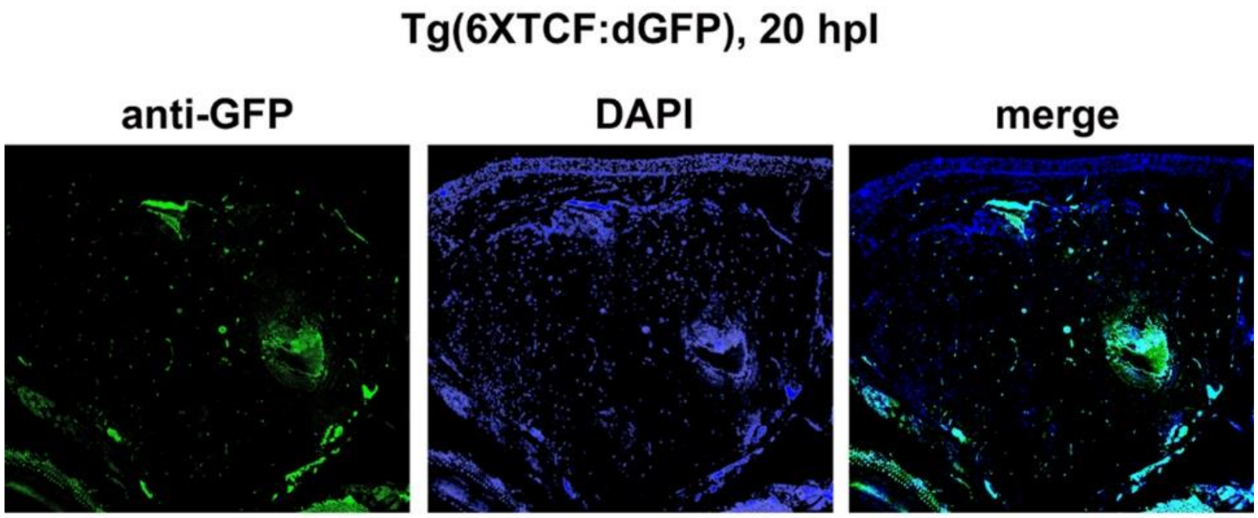

Supplementary Figure 7: Immunofluorescence staining for anti-GFP in Tg(6XTCF:dGFP) transgenic zebrafish reporter of Tcf/Lef-mediated transcription at 20 hpl. GFP expression is visible around the lesioned area, most likely in the endothelial and blood cells of the brain.

Demirci, Cucun et al., Figure S8

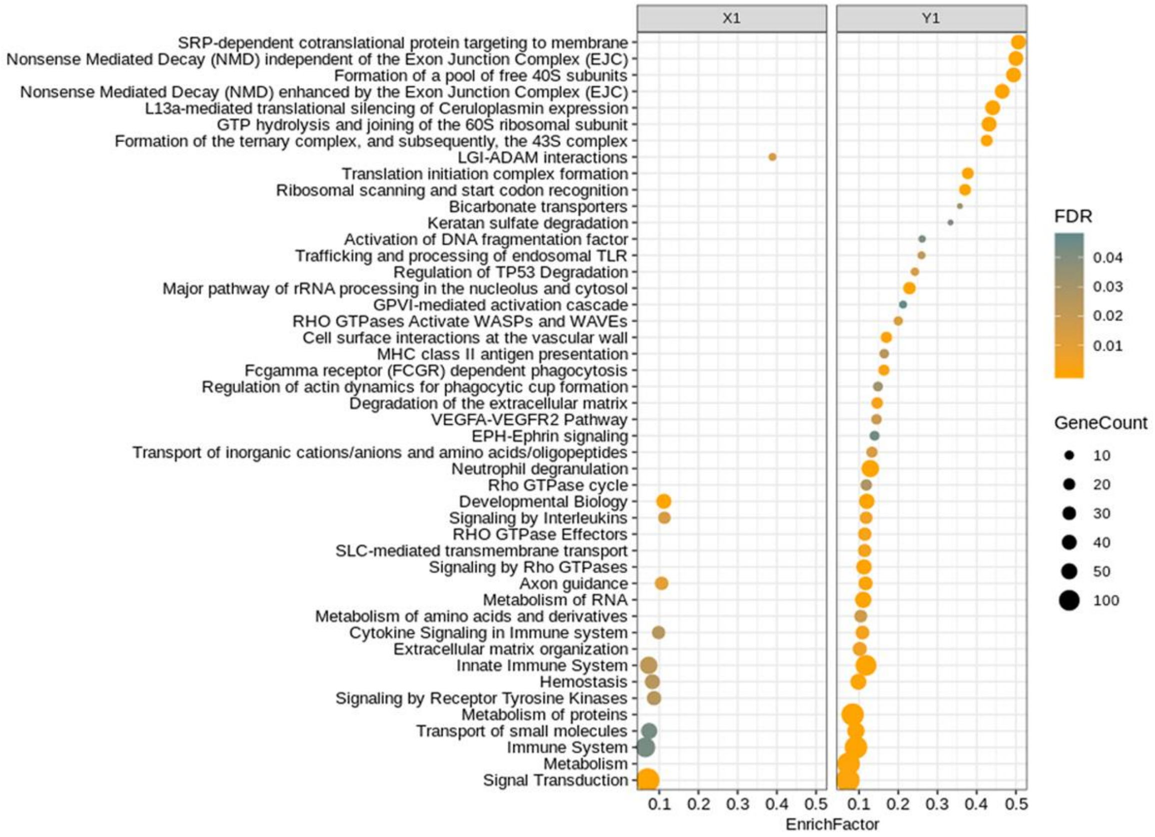

Supplementary Figure 8. Reactome pathways altered in lesioned and unlesioned hemispheres at the early wound healing and proliferative stages of brain regeneration by using all significantly changed genes STRING bioinformatics tool was used to show the most significantly enriched Reactome pathways based on the transcriptional changes in X1 and Y1. Dot plot represents Reactome pathways enriched in Y1 and Y2 by using all significantly changed genes (X1 1472, Y1 1707) in these comparisons based on FDR and EnrichFactor. STRING: Search Tool for the Retrieval of Interacting Genes/Proteins, FDR: false discovery rate. See Materials and Methods for definition of DEGs covered in X and Y comparisons.

Demirci, Cucun et al., Figure S9

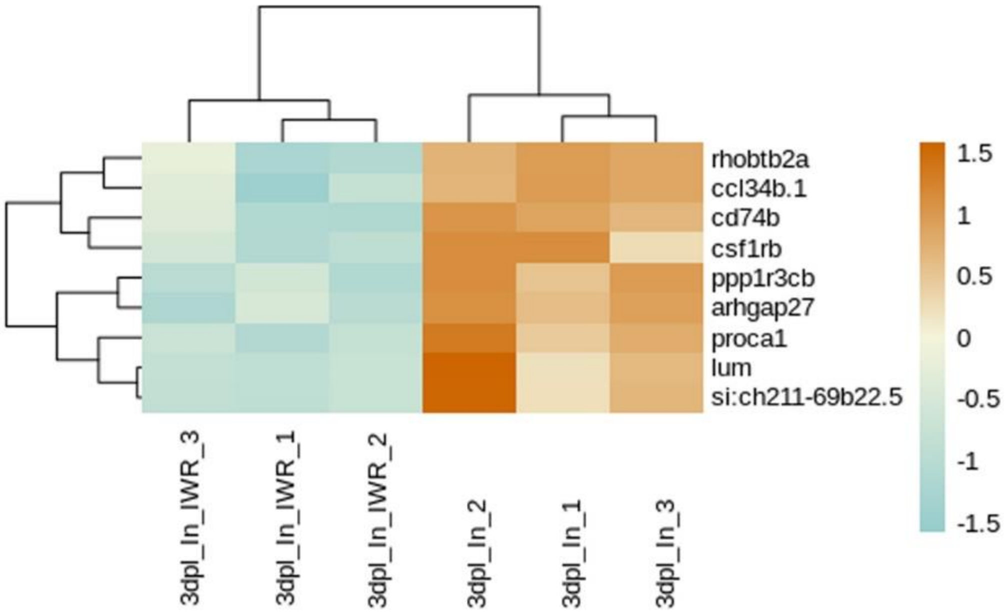

Supplementary Figure 9. The Wnt targetome in the proliferative stage of brain regeneration Intersection heatmap shows Wnt target genes that are Up in Y1 and Down in Y3. Each column represents a single hemisphere from a single telencephalon and each row shows a single gene. The scale bar shows Z-scores for the heatmaps from high to low expression, represented by a color gradient from orange to green, respectively.

Demirci, Cucun et al., Figure S10

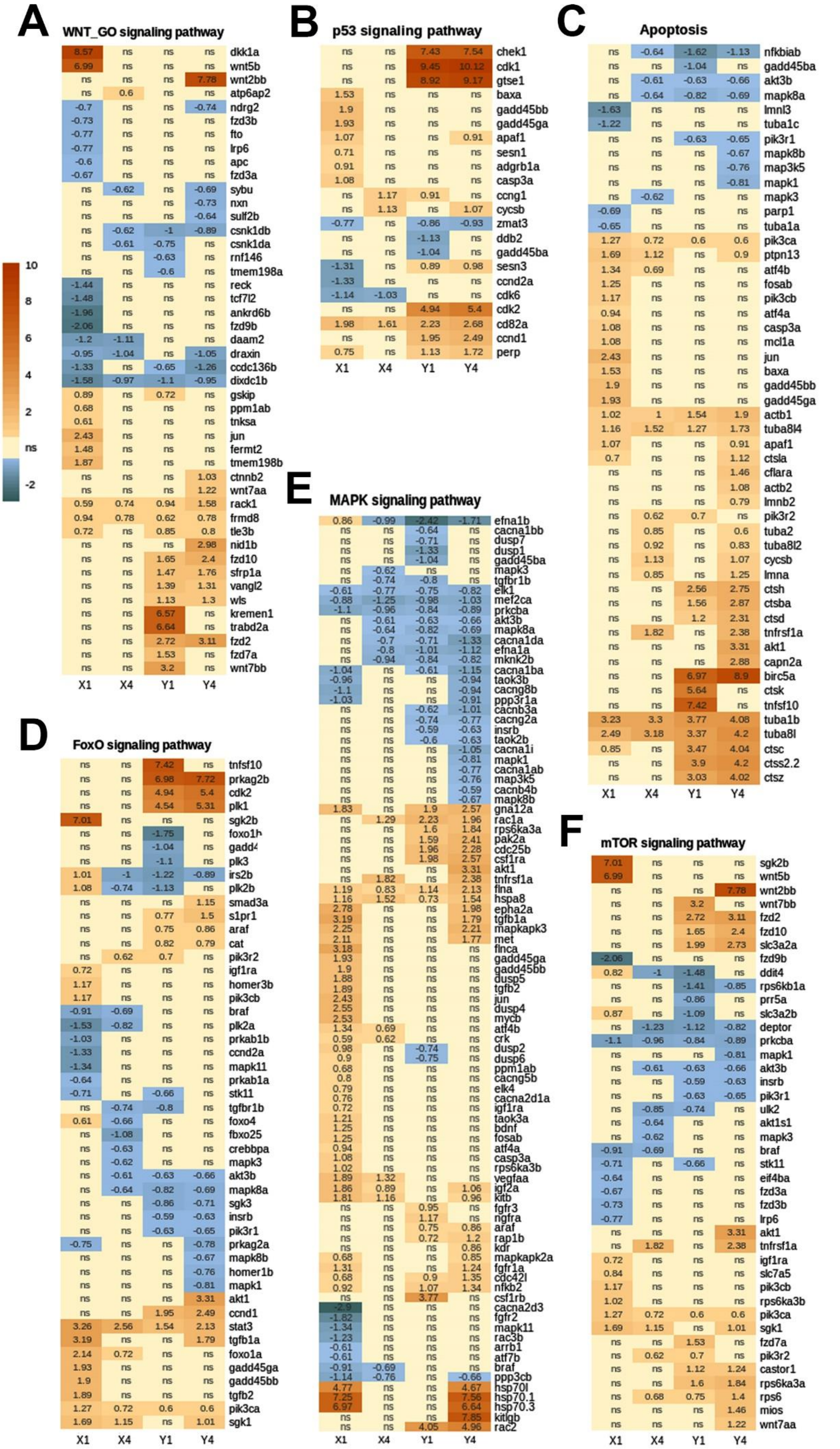

Supplementary Figure 10. Various KEGG pathways are transcriptionally altered during early wound healing and proliferative stages of brain regeneration in response to inhibition of Wnt/β-catenin signaling Heatmap representation of uniquely changed genes in (A) WNT GO term, (B) p53 signaling pathway, (C) apoptosis pathway, (D) FoxO signaling pathway, (E) MAPK signaling pathway and (F) mTOR signaling pathway in X1, X4, Y1 and Y4 comparisons. Each column represents a comparison according to DESeq2 results and each row shows a single gene. The scale bar shows log_2 of the fold change values for the heatmaps from high to low expression, represented by a color gradient from orange to blue, respectively. Statistical significance was evaluated using the Wald test. ns: non-significant. See Materials and Methods for definition of DEGs covered in X and Y comparisons.

Supplementary Results and Discussion

Transcriptome profiling of the telencephalon during early wound healing stage of regeneration

Based on GO term analysis of Up and Down genes separately, DEGs in X1 were enriched in a total of 95 terms in group BP, 21 in group CC and 79 in group MF, while DEGs in X2 were enriched in 17 terms in group BP, 8 in group CC and 15 in group MF (Table S4). The top 40 GO terms in X1 (10 Down and 10 Up in BP, 5 Down and 5 Up in CC and 5 Down and 5 Up in MF) and top 26 GO terms in X2 (10 Down and 2 Up in BP, 2 Down and 5 Up in CC and 5 Down and 2 Up in MF) showed no overlap, parallel to the low number of genes shared between X1 and X2 (Figure 2D-E). We also identified the Reactome pathways significantly enriched in X1 and X2, which showed markedly lower gene counts as compared to X1 (Figure S3, Table S5).

Transcriptome profiling of the telencephalon during early proliferative stage of regeneration

GO term enrichment analysis of Up and Down genes separately revealed that DEGs in Y1 were enriched in 110 terms in group BP, 32 in group CC and 63 in group MF, while DEGs in Y2 were enriched in 58 terms in group BP, 15 in group CC and 42 in group MF (Table S4). The top 40 GO terms in Y1 and Y2 (10 Down and 10 Up in BP, 5 Down and 5 Up in CC and 5 Down and 5 Up in MF) overlapped by 25% (Figure 3D-E). GO terms were also defined for all significantly changed DEGs in Y1 and Y2 groups (Figure S4, Table S4). Although there was no KEGG pathway enriched in Y2, we determined 15 KEGG pathways significantly enriched in Y1 (Figure 3F, Table S5). We found 44 and 7 Reactome pathways to be enriched in Y1 and Y2, respectively (Figure S5, Table S5).

Early wound healing and proliferative stages of brain regeneration are more different than similar

DEGs unique to X1 were enriched in a total of 83 GO terms (50 BP, 5 CC and 28 MF) while DEGs in group 2 were enriched in 58 GO terms (40 BP, 12 CC and 23 MF) (Table S4). These GO terms were only enriched

for DEGs that are unique to X1, but not for DEGs that are unique to Y1 or DEGs shared between X1 and Y1. We found 11 and 44 Reactome pathways that were enriched in X1 and Y1, respectively (Figure S8, Table S5). Interestingly, none of the DEGs we detected at either early wound healing or proliferative stage were regulated oppositely in the two hemispheres, i.e. these genes were either upregulated or downregulated in both halves. At 20 hpl, the number of DEGs in the lesioned hemisphere (X1) is far higher than that of the unlesioned one (X2), whereas at 3 dpl, the number of DEGs in the corresponding halves (Y1 and Y2) both increases and converges. However, the higher number of DEGs at 3 dpl is not reflected in the number of KEGG pathways enriched at this stage, that is the number of KEGG pathways in Y1:Y2 (15:0) was fewer than in X1:X2 (20:4) (see Figures 2F, 3F). Strikingly, the number of DEGs that are shared between X1 and X2 is far fewer than between Y1 and Y2, which shared half of their DEGs including the most strongly downregulated five genes in both groups. This finding is further supported by the low number of DEGs shared between X1 and Y1.

A noteworthy point is that some of the very strongly altered DEGs are shared between the lesioned and unlesioned hemispheres even at different stages of telencephalon regeneration. *en1a*, encoding for the homeobox transcription factor Engrailed-1a, stands out as a notable example of one of the most strongly downregulated genes in X1, Y1 and Y2 (see Table S2). The mouse ortholog *En1* is involved in development and maintenance of the monoaminergic structures such as dopaminergic and serotonergic circuitries in the mid- and hindbrain (Fox and Deneris, 2012; Kouwenhoven et al., 2016). More interestingly, the latter work has described an additional role for En1 as suppressor of Wnt1 and canonical Wnt-signaling in the hindbrain (Kouwenhoven et al., 2016). Thus, strong suppression of *en1a* may indicate loss of ventral dopaminergic neurons, leading to elevation of Wnt/β-catenin signaling. *entpd2b* gene, which is strongly upregulated at 20 hpl and 3 dpl, has been shown to be also upregulated after hepatocellular injury (Feldbrugge et al., 2018), suggesting that it undertakes regeneration-promoting roles in different cellular contexts.

Inhibition of Wnt/β-catenin signaling during early wound stage results in a marked alteration of gene expression profiles represented in KEGG pathway enrichment The genes we obtained from KEGG database included 189 genes involved in Wnt signaling pathway, 371 in MAPK signaling pathway, 167 in Apoptosis, 187 in mTOR signaling pathway, 79 in p53 signaling pathway and 167 in FoxO signaling pathway (Table S8). Besides, GO term enrichment analysis using AmiGO database revealed 275 genes related to Wnt signaling (Table S8). The number of significantly altered genes in X1 decreased dramatically after IWR treatment in X4 as follows: Wnt 21→6, Wnt-GO 25→9, p53 13→4, apoptosis 23→17, FoxO 23→16, MAPK 56→22 and

mTOR 18→13 (Figures 7A, 7C-D, S10 compare the total number of brown and blue shaded boxes in X1 and X4; Table S8). 11/13 DEGs detected in p53 signaling, 16/23 in apoptosis, 17/23 in FoxO signaling, 44/56 in MAPK pathway and 14/18 in mTOR signaling were not any more differentially expressed or regulated in the opposite direction, indicating a regulatory role for Wnt/β-catenin signaling in the early stage of brain regeneration (Figures S10B-F). On the contrary, the number of altered genes in Y1 either did not change much or even increased in Y4 as follows: Wnt 17→20, Wnt-GO 19→20, p53 12→11, apoptosis 20→32, FoxO 23→23, MAPK 37→52 and mTOR 21→20 (Figures 7A, S10 compare the total number of brown and blue shaded boxes in Y1 and Y4; Table S8). The weak response to Wnt pathway in the early proliferative stage of regeneration is further supported by the fact that there was no change in gene expression pattern of 9/12 DEGs detected in p53 signaling, 16/20 in apoptosis, 15/23 in FoxO signaling, 27/37 in MAPK pathway and 13/21 in mTOR pathway after IWR treatment in Y4 (Figure S10B-F).

Since canonical Wnt activity returned to control levels at the proliferative phase of regeneration, RGC proliferation appears to be regulated via another signaling pathway such as Notch, whose inhibition has been shown to cause a reduction in the number of RGCs in the lesioned hemisphere of the telencephalon (Chapouton et al., 2010; Kishimoto et al., 2012; de Oliveira-Carlos et al., 2013). Such a regulatory role in proliferative response is strongly supported by our findings showing that *notch2*, *notch3*, *notch1b* are exclusively upregulated in the lesioned half of the telencephalon at 3 dpl.

Supplementary References

Chapouton, P., Skupien, P., Hesl, B., Coolen, M., Moore, J.C., Madelaine, R., et al. (2010). Notch activity levels control the balance between quiescence and recruitment of adult neural stem cells. *J Neurosci* 30(23), 7961-7974. doi: 10.1523/JNEUROSCI.6170-09.2010.

de Oliveira-Carlos, V., Ganz, J., Hans, S., Kaslin, J., and Brand, M. (2013). Notch receptor expression in neurogenic regions of the adult zebrafish brain. *PLoS One* 8(9), e73384. doi: 10.1371/journal.pone.0073384.

Feldbrugge, L., Jiang, Z.G., Csizmadia, E., Mitsuhashi, S., Tran, S., Yee, E.U., et al. (2018). Distinct roles of ecto-nucleoside triphosphate diphosphohydrolase-2 (NTPDase2) in liver regeneration and fibrosis. *Purinergic Signal* 14(1), 37-46. doi: 10.1007/s11302-017-9590-3.

Fox, S.R., and Deneris, E.S. (2012). Engrailed is required in maturing serotonin neurons to regulate the cytoarchitecture and survival of the dorsal raphe nucleus. *J Neurosci* 32(23), 7832-7842. doi: 10.1523/JNEUROSCI.5829-11.2012.

Kishimoto, N., Shimizu, K., and Sawamoto, K. (2012). Neuronal regeneration in a zebrafish model of adult brain injury. *Dis Model Mech* 5(2), 200-209. doi: 10.1242/dmm.007336.

Kouwenhoven, W.M., Veenvliet, J.V., van Hooft, J.A., van der Heide, L.P., and Smidt, M.P. (2016). Engrailed 1 shapes the dopaminergic and serotonergic landscape through proper isthmic organizer maintenance and function. *Biol Open* 5(3), 279-288. doi: 10.1242/bio.015032.
